# Genome-wide detection of CNVs in Chinese indigenous sheep with different types of tails using ovine high-density 600K SNP arrays

**DOI:** 10.1038/srep27822

**Published:** 2016-06-10

**Authors:** Caiye Zhu, Hongying Fan, Zehu Yuan, Shijin Hu, Xiaomeng Ma, Junli Xuan, Hongwei Wang, Li Zhang, Caihong Wei, Qin Zhang, Fuping Zhao, Lixin Du

**Affiliations:** 1 National Center for Molecular Genetics and Breeding of Animals, Institute of Animal Sciences, Chinese Academy of Agricultural Sciences, Beijing 100193, China; 2 College of Animal Science and Technology, China Agricultural University, Beijing 100193, China; 3 College of Animal Science and Technology, Gansu Agricultural University, Lanzhou 730070, China; 4 Beijing Compass Biotechnology Co., Ltd., Beijing 100192, China

## Abstract

Chinese indigenous sheep can be classified into three types based on tail morphology: fat-tailed, fat-rumped, and thin-tailed sheep, of which the typical breeds are large-tailed Han sheep, Altay sheep, and Tibetan sheep, respectively. To unravel the genetic mechanisms underlying the phenotypic differences among Chinese indigenous sheep with tails of three different types, we used ovine high-density 600K SNP arrays to detect genome-wide copy number variation (CNV). In large-tailed Han sheep, Altay sheep, and Tibetan sheep, 371, 301, and 66 CNV regions (CNVRs) with lengths of 71.35 Mb, 51.65 Mb, and 10.56 Mb, respectively, were identified on autosomal chromosomes. Ten CNVRs were randomly chosen for confirmation, of which eight were successfully validated. The detected CNVRs harboured 3130 genes, including genes associated with fat deposition, such as *PPARA, RXRA, KLF11, ADD1, FASN, PPP1CA, PDGFA*, and *PEX6*. Moreover, multilevel bioinformatics analyses of the detected candidate genes were significantly enriched for involvement in fat deposition, GTPase regulator, and peptide receptor activities. This is the first high-resolution sheep CNV map for Chinese indigenous sheep breeds with three types of tails. Our results provide valuable information that will support investigations of genomic structural variation underlying traits of interest in sheep.

Chinese indigenous sheep breeds can be classified into fat-tailed, fat-rumped, and thin-tailed sheep breeds. Worldwide, more than 25% of sheep breeds are fat-tailed or fat-rumped and store large amounts of fat in the tail^1^. It is thought that fat-tailed sheep, which appeared during the process of domestication, are the result of breeding by humans and natural selection^2^. Fat-tailed sheep were first documented approximately 5000 years ago^2^. The tails of fat-tailed and fat-rumped sheep are considered to be adaptations to hostile environments and act as energy reserves to support migration and survival during cold winters^3^. In addition, the fat in the tails of fat-tailed and fat-rumped sheep can be used by humans as a high-energy food during periods of drought and famine^2^,^4^. Therefore, natural and artificial selection has led to an increased prevalence of fat in sheep tails across generations^3^,^4^.

Copy number variation (CNV), a type of genomic structural variation, refers to DNA segments with sizes ranging from 1 kilobase (Kb) to several megabases (Mb) in which duplication or deletion events have occurred^5^. CNVs are ubiquitously distributed in the human genome and can influence phenotypic variation via changes in gene expression and gene dosage^6^,^7^,^8^. CNV is a major source of genetic variation and phenotypic diversity^8^,^9^. CNV in the *ASIP* gene determines the white and grey coat phenotypes in goats^10^. In addition, CNV in the *KIT* gene leads to a white coat colour in pigs^11^, whereas the phenotype of the pea comb in chickens is influenced by CNV in intron 1 of *SOX5*^12^. Chinese indigenous sheep breeds show tremendous phenotypic differences, especially in the tail. Large-tailed Han sheep have the largest and fattiest tail of any Chinese sheep breed, characterized by being long, broad, and fat tail with a thin, twisted end turned upwards between two lobes and a broad base. Altay sheep have fat deposits in the buttocks that look like a tail; thus, Altay sheep are known as fat-rumped sheep, while Tibetan sheep have a thin tail the shape of a flattened cone^13^. To understand the genetic basis of these three types of tails, we aimed to identify the potential contribution of CNV to phenotypic variation.

Therefore, the primary aim of this study is to detect CNV in Chinese indigenous sheep breeds with different types of tails. Genome-wide CNV detection was carried out by high-density 600K SNP genotype arrays. The PennCNV program was used to analyse the data and validate the CNVRs by qPCR. To the best of our knowledge, this is the first sheep CNVR map constructed based on high-density 600K SNP genotype data. We also compared our results with those from previous studies of sheep CNV. This study provides a comprehensive map of CNV in the ovine genome that will guide studies aimed at elucidating the causes of complex traits.

## Results

### SNP genotyping

A total of 120 sheep were genotyped using the Illumina Ovine SNP 600K BeadChip. Samples with low-quality signal intensity were excluded based on the CNV quality control filter criteria. A total of 110 individuals from three sheep breeds (36 large-tailed Han sheep, 37 Altay sheep, and 37 Tibetan sheep) were analysed.

### Genome-wide detection of CNVs in sheep

**Figure 1:**
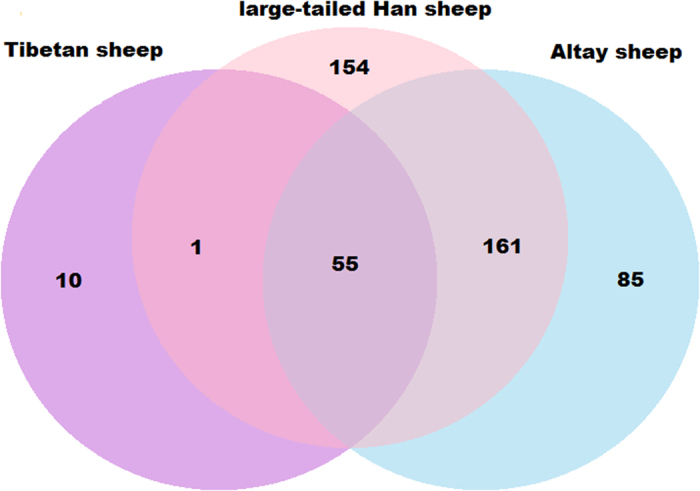
Numbers of CNVRs identified among sheep breeds with three different types of tails and the numbers of CNVRs overlapping between different breeds.

**Figure 2:**
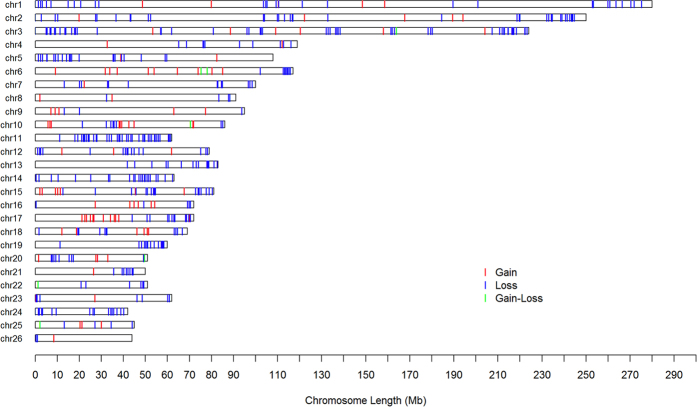
Genomic distribution and status of detected CNVRs among sheep breeds with three different types of tails. Red, green, and blue lines represent the predicted statuses of gain, loss, and gain or loss, respectively.

Table 1 summarizes the initial numbers of CNVs and CNVRs as identified by PennCNV software in the three selected breeds. After merging overlapping CNVs, a total of 371, 301, and 66 CNVRs with lengths of 71.35 Mb, 51.65 Mb, and 10.56 Mb were detected on 26 pairs of autosomal chromosomes in large-tailed Han sheep, Altay sheep, and Tibetan sheep, respectively (Supplementary File 1: Tables S1–S3). A number of differences were found in the numbers of CNVRs among the three breeds. A total of 216 CNVRs were shared by large-tailed Han sheep and Altay sheep, 56 CNVRs shared by large-tailed Han sheep and Tibetan sheep, 55 CNVRs shared by Altay sheep and Tibetan sheep, and 55 CNVRs shared by all three breeds, as shown in Fig. 1. A CNV map of sheep with different types of tails was constructed by removing repetitive CNV regions, as shown in Fig. 2. CNVRs were not uniformly distributed across chromosomes. Among the 490 CNVRs, 93 were gains, 390 were losses, and 7 were losses and gains within the same region. The length of the CNVRs ranged from 100.11 to 804.18 Kb, with an average length of 165.39 Kb and a median length of 133.17 Kb (Supplementary File 1: Table S4). The ratio of CNVRs on 26 pairs of autosomal chromosomes ranged from 1.16% to 82.50%. Chromosome 3 had the most CNVRs (42), as shown in Table 2. No CNVRs were identified on chromosome 25 or 26 in Tibetan sheep (Supplementary File 2: Fig. S1).

### CNV Validation by qPCR

**Figure 3:**
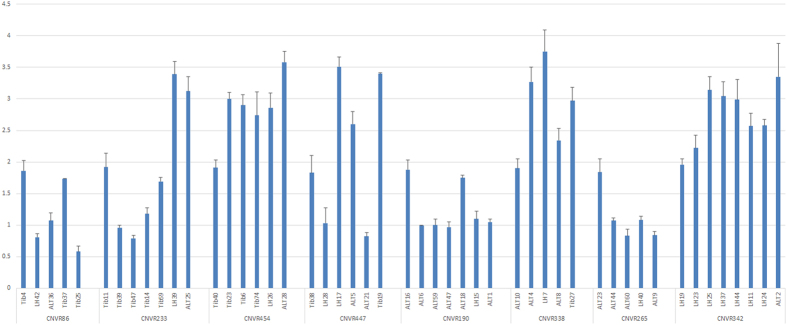
Normalized ratios (NRs) obtained by QPCR for 8 CNVRs. The *y*-axis shows the NR values obtained by QPCR, and the *x*-axis shows the sample names in the different CNV regions. Samples with NRs of approximately 2 denote normal individuals (two copy), samples with NRs of approximately 1 denote one-copy-loss individuals (one copy), and samples with NRs of approximately 3 or more denote copy-number-gain individuals.

To confirm the identified CNVRs, we randomly chose 10 CNVRs for validation by qPCR. The 10 selected CNVRs represented different types of CNV (gain, loss, and gain/loss) and were chosen from all three of the tested sheep breeds. Eight (80%) of the randomly selected CNVRs were confirmed in agreement with PennCNV (Fig. 3).

### Gene content of sheep CNVRs

In large-tailed Han sheep, a total of 2881 annotated genes within 329 CNVRs (88.67%) were identified from the Ensembl Genes 64 Database using the BioMart data management system, while the remaining 42 CNVRs (11.32%) lacked annotated genes. Within the 301 CNVRs that were detected in Altay sheep, 244 CNVRs (81.06%) harboured 1795 annotated genes, whereas the other 57 CNVRs (18.94%) did not contain annotated genes. In Tibetan sheep, 293 annotated genes were contained within 60 CNVRs (90.90%), whereas the remaining 6 CNVRs (9.09%) lacked annotated genes. A total of 1839 common genes were shared among large-tailed Han sheep, Altay sheep, and Tibetan sheep. Therefore, a total of 3130 genes resided in the detected CNVRs.

To provide insight into the functions of the genes in the identified CNVRs, GO and KEGG pathway analyses were performed using the DAVID tool. After the conversion of *Ovis aries* Ensembl gene IDs to human orthologue Ensembl gene IDs using BioMart, 3016 corresponding human gene IDs remained in DAVID. ‘Human’ was selected as the background for subsequent analysis. After Bonferroni correction, 50 GO terms were identified as significantly enriched (Supplementary File 2: Table S5). The significantly enriched GO terms were involved in thyroid hormone receptor activator activity (GO: 0010861) and coactivator activity associated with energy expenditure (GO: 0030375)^14^. In addition, some significantly enriched GO terms were involved in GTPase regulator activity (GO: 0030695), GTPase activator activity (GO: 00005096), regulation of GTPase activity (GO: 0043087), regulation of *Ras* protein signal transduction (GO: 0046578), skeletal system development (GO: 0001501), embryonic morphogenesis (GO: 0048706), embryonic limb morphogenesis (GO: 0048598), transcription regulator activity (GO: 0030528), and other biological processes. KEGG pathway analysis revealed that the genes in the identified CNVRs were enriched in 2 significant pathways: ribosome and notch signalling (Supplementary File 2: Table S6).

### Difference between fat-tailed and thin-tailed sheep breeds

Among the breeds that were selected for this study, large-tailed Han sheep and Altay sheep are fat-tailed and fat-rumped sheep, respectively, while Tibetan sheep are thin-tailed sheep. There were many differences in the CNV of fat-tailed and thin-tailed sheep. Fat-tailed sheep harboured 211 CNVRs covering 56 Mb of the sheep genome sequence and 123 genes. After GO enrichment analysis, some genes associated with fat were identified (Table 3). Seven genes associated with fat were identified in the CNVRs of large-tailed Han sheep, including peroxisome proliferator-activated receptor-α (*PPARA*), retinoic X receptor A (*RXRA*), Kruppel-like factor 11 (*KLF11*), adipocyte determination and differentiation factor 1 (*ADD1*), fatty acid synthase (*FASN*), phosphoprotein phosphatase 1 catalytic subunit A (*PPP1CA*), and platelet-derived growth factor alpha (*PDGFA*). In Altay sheep, 5 genes in CNVRs were associated with fat deposition, including peroxin 6 (*PEX6*), *RXRA*, *FASN*, *PPP1CA*, and *PDGFA*. Tibetan sheep are thin-tailed sheep native to a high-altitude plateau. Sixty-six CNVRs were identified in Tibetan sheep; however, no CNVRs were detected on chromosome 25 or 26. In Tibetan sheep, only 1 gene related to fat deposition was found within the identified CNVRs: *RXRA*. Two genes related to adaptation to high altitude were found within the identified CNVRs in Tibetan sheep: α-ketoglutarate-dependent dioxygenase alkB homologue 5 (*ALKBH5*) and nuclear prelamin A recognition factor-like (*NARFL*).

### Comparison with other studies on CNV in sheep

Our results were compared to those of previous reports on sheep genomic CNV. The first study on sheep genome CNV was conducted by Fontanesi *et al*.^15^ using a tiling oligonucleotide array with approximately 385,000 probes that was used to detect 135 CNVRs from 11 ewes belonging to 6 different Italian dairy or dual-purpose sheep breeds. Eleven of the 135 CNVRs identified were also identified in our results (Supplementary File 1: Table S7, Table 4). Liu^16^ identified 238 CNVRs by assessing 329 individuals from 3 breeds using the Ovine SNP 50 BeadChip, but only 12 of these CNVRs on 10 chromosomes were also identified in our results (Supplementary File 1: Table S8). The most recent study on CNV in sheep was carried out by Ma^17^, who identified 111 CNVRs by subjecting 160 individuals from 8 breeds to analysis using the Ovine SNP 50 BeadChip; only 8 of these CNVRs on 4 chromosomes were also identified in our results (Supplementary File 1: Table S9).

## Discussion

In recent years, with the development of high-throughput genotyping technology, CNV detection using SNP chip data has been conducted in humans and animals^18^,^19^,^20^. Several algorithms for inferring CNV based on SNP chip data have been developed, including PennCNV, cnvPartition, and QuantiSNP. No single approach can capture all CNV; thus, one may be complementary to another. However, most studies on CNV using SNP arrays in animals and humans have used only PennCNV software^21^,^22^,^23^, especially for high-density SNP data^22^,^23^,^24^,^25^,^26^,^27^,^28^. PennCNV detects CNV more reliably than do some other algorithms^29^ because PennCNV can incorporate multiple sources of information, including total signal intensity and allelic frequency SNPs^30^,^31^. When multiple algorithms are used to detect CNV, determining the appropriate number of CNVs may be difficult. If we accept only CNVs that are commonly detected by all algorithms, many CNVs are missed, but many false-positive CNVs are identified if we accept all CNVs detected by different algorithms. Finally, high-density SNP chips and stricter filtering criteria could ensure a high positive validation rate. Although we used only PennCNV to detect CNVs, two strict criteria were adopted to reduce the risk of false-positive results: SD of LRR < 0.30 and BAF = 0.01. These strict criteria and high-density SNP array data likely resulted in the high qPCR confirmation percentage of 80% in the present study. In our study, most of the predicted positive samples identified by the PennCNV program agreed well with the qPCR experiments and showed a high level of copy number concordance between them. The discrepancy between PennCNV and qPCR findings may indicate uncertain boundaries of CNV based on the SNP array, as well as the potential impacts of SNPs and small indels.

It is notable that few of the CNVRs that were identified in this study were identified in other sheep CNV studies, similar to the results of other CNV studies in humans and mammals^26^,^36^. The inconsistency between results from different studies might be due to the following reasons: (1) different genetic backgrounds and sample sizes; (2) different chip platforms (Illumina SNP chips or CGH arrays); and (3) SNP chips with different densities and CNV-calling algorithms.

In this study, we selected three sheep breeds distributed in different parts of China with obviously different types of tails: Altay sheep, large-tailed Han sheep and Tibetan sheep. Altay sheep live in the Gobi Desert, where the annual average temperature and extreme minimum temperature are 4.0 °C and −42.7 °C, respectively, and the ground is snow-covered for 200–250 days of the year. In such an environment, fat tends to be deposited on the rump of the animal, forming a substantial buttock that is an adaptation to the harsh environment of the Gobi Desert. Our results indicate that the process of adaptation to extreme climates in Altay sheep is principally mediated through complex, integrated metabolic responses, as has been confirmed in rodents^33^. Climate has a significant impact on animal fitness and physiology, especially in ruminants^34^. Climate factors, including solar radiation, temperature, UV radiation, humidity, and precipitation, have direct impacts on sheep; some of these climate factors, such as temperature and precipitation, also directly affect the quality, quantity, and digestibility of food, thus impacting sheep indirectly^35^. Therefore, long-term thermal stress can lead to energy metabolic adaptation, as well as cold and heat tolerance, in particular breeds^36^. At the same time, sheep with different types of tails (fat-tailed and thin-tailed) also follow basic thermoregulatory principles to conserve or dissipate body energy during different seasons. The ability to deposit fat in the tail is beneficial in extremely harsh environments and seasons. Large-tailed Han sheep are primarily produced in the hinterland of the northern Chinese plain, which has clear seasonal variation, with cold, dry winters and hot, rainy summers. We found some candidate genes involved in endocrine regulation, consistent with the impact of day length and seasonal timing on sheep physiology and evolutionary fitness. The tails of large-tailed Han sheep show hypertrophy and have a fan-like shape, sagging below the hock and dragging on the ground in some individuals. During periods of famine, the tails of fat-tailed sheep can be used as food.

Thin-tailed Tibetan sheep live in the mountainous region of the Tibetan plateau at an altitude of 3000–5000 metres. Tibetan sheep are relatively strong and tall, with a thin tail in the shape of a flat cone. We found that fat-tailed and fat-rumped sheep had more CNVRs (371 and 301, respectively) than did thin-tailed sheep (66). In the CNVRs of fat-tailed sheep, we detected some genes associated with fat. In the CNVRs of thin-tailed sheep, we found two genes (*ALKBH5* and *NARFL*) associated with altitude adaptation. It is generally believed that the first wild sheep were thin-tailed sheep, from which fat-tailed sheep were produced as the result of selection by humans and by nature^37^. These results indicate that natural and artificial selection for fat deposition in sheep tails may lead to CNV alterations.

In this study, we identified genes in CNVRs associated with fat synthesis and metabolism in fat-tailed (7 genes), fat-rumped (5 genes), and thin-tailed (1 gene) sheep. *RXRA* was identified in CNVRs in all three types of sheep, indicating that it may play an important role in the maintenance of basic fat metabolism. *PPARA*, *RXRA*, *KLF11*, *ADD1*, *FASN*, *PPP1CA*, *PDGFA*, and *PEX6* were only identified in fat-tailed and fat-rumped sheep, indicating that these genes may cause tail fat deposition. *PPARA* is a member of the *PPAR* family. Peroxisomes play an important role in fatty acid metabolism. Unsaturated fatty acids bind to *PPARA*, which is highly expressed in the liver, skeletal muscle, heart, and kidney^38^, where it activates genes that are involved in fatty acid metabolism. *RXRA* is a member of the nuclear hormone superfamily and forms heterodimers with a number of nuclear receptors, including the thyroid hormone receptor, vitamin D receptor, *GR*, and *PPAR*^39^. *RXRA* can mediate heterodimerization with retinoic acid receptors (*RARs*) and is essential for the functional activation of *RARs* by their ligands^40^. *RXRA* plays a role in lipid homeostasis^41^,^42^ and foetal development^39^. *KLF11* (Kruppel-like factor 11) is a member of the Kruppel-like factor family and is expressed in many human tissues^43^. Loft^44^ reported that *KLF11* is a browning transcription factor in human adipocytes. *PPAR* directly induces *KLF11* and, together with *KLF11*, activates and maintains the brite-selective gene programme. *ADD1* (adipocyte determination- and differentiation-dependent factor 1), a member of the basic helix-loop-helix (*bHLH*) family of transcription factors, is associated with adipocyte differentiation and cholesterol homeostasis^45^. *ADD1* plays an important role in the process of adipocyte differentiation^46^, as well as in the activation of *PPAR* and *C/EBPs*. *FASN* (fatty acid synthase) plays a key role in the de novo synthesis of fatty acids in mammals. *FASN* catalyses reaction steps in the conversion of acetyl-CoA, malonyl-CoA, and NADPH into saturated fatty acids. *FASN* is expressed in the mammalian liver and adipose tissue, where it regulates body fat deposition and fatty acid anabolism^47^. *PPP1CA* (phosphoprotein phosphatase 1 catalytic subunit alpha isozyme) is an isoform of *PPP1C*^48^, which was first identified as the enzyme that converts phosphorylase A into phosphorylase B^49^. A number of roles have been attributed to *PPP1C* in the regulation of important cellular events. *PDGFA* (platelet-derived growth factor A) is a *PDGF* subunit^50^. *PDGF* has been directly implicated in developmental and physiological processes^51^. *PEX6* (peroxin 6) is a member of the *PEX* family. Peroxidase is involved in fatty acid metabolism, glycometabolism, toxic material degradation, and oxygen concentration regulation. The biosynthesis and proliferation of the peroxisome are regulated by *PPARs* and *PEX*. Because super-long-chain fatty acids cannot enter mitochondria^52^, they must be oxidized into short fatty acids by peroxidase. Some of the differences may be due to selection because of different environments. We also identified Rab family protein *TBC1D13*, a GTPase activator that plays an important role in increasing GTPase activity^53^. GTP hydrolysis is catalysed by GTPases, after which GDP serves as an energy source in metabolic reactions within the cell.

To better understand the molecular function of these candidate genes, we examined their GO classification. Many of the genes that were detected in our study were consistent with prior expectations because they are involved in adipose tissue energy balance, skeletal system development, and metabolic activities. Two intriguing candidate GO terms were thyroid hormone receptor activator activity (GO: 0010861) and coactivator activity (GO: 0030375) related to energy expenditure. These observations should be explored and verified in future studies.

## Conclusions

We performed genome-wide CNV detection based on ovine high-density genotyping data from 120 sheep with three types of tails and provide the highest resolution CNV map of the sheep genome produced to date. Among the 490 CNVRs, 93 were gains, 390 were losses, and 7 were losses and gains within the same region. The length of the CNVRs ranged from 100.11 to 804.18 Kb, with an average length of 165.39 Kb and a median length of 133.17 Kb. A total of 371, 301, and 66 CNVRs were identified in large-tailed Han sheep, Altay sheep, and Tibetan sheep, respectively. The CNVR regions harboured genes associated with fat deposition and fat synthesis in fat-tailed sheep and fat-rumped sheep. The CNVR regions harboured genes associated with adaptation to the low oxygen environment of the Tibetan plateau in Tibetan sheep. These candidate genes included *PPARA, RXRA, KLF11, ADD1, FASN, PPP1CA, PDGFA*, and *PEX6*. Moreover, multilevel bioinformatics analyses of the detected candidate genes showed that they were likely involved in fat deposition, GTPase regulation, and peptide receptor activities. This study is an important step towards the generation of a CNV map of the ovine genome and provides an important resource for studies of ovine genomic variation. In addition, our findings provide valuable information that will facilitate studies investigating genomic structural variation underlying traits of interest in sheep, including types of sheep tails, fat deposition in sheep tails, and environmental adaptability.

## Materials

### Ethics Statement

All of the animal procedures were performed in strict accordance with the guidelines proposed by the China Council on Animal Care and the Ministry of Agriculture of the People’s Republic of China. All of the animal experiments were approved by the Chinese Academy of Agricultural Sciences (CAAS) (Beijing, China).

### DNA sample collection

For this study, 120 individuals from 3 breeds, including 40 large-tailed Han sheep (20 rams and 20 ewes), 40 Altay sheep (20 rams and 20 ewes), and 40 Tibetan sheep (20 rams and 20 ewes), were collected from Liaocheng in Shandong Province, Altay in Xinjiang Province, and Tianzhu in Gansu Province, respectively. All of the specimens were randomly selected.

Genomic DNA samples were extracted from blood using the TIANamp Blood DNA Kit (Tiangen Biotech Co. Ltd., Beijing, China). The purity and concentration of genomic DNA were measured using a NanoVue Spectrophotometer.

### Genotyping and quality control

The genomic DNA of each specimen was genotyped with the Illumina Ovine SNP 600 BeadChip (Illumina Inc., CA, USA), which contains 604,715 SNPs spanning the ovine genome with an average distance of 4.28 Kb.

To increase the accuracy of CNV inference, stringent quality control criteria were applied: (1) individual call rate >95% and call frequency >90%; (2) LRR standard deviation <0.30; (3) BAF drift <0.01; and (4) default waviness factor.

### Detection of CNV

Similar to previous studies^27^, chromosomes X and Y were excluded from the analysis. PennCNV software was only used to detect CNV on autosomes and was downloaded from http://penncnv.openbioinformatics.org/en/latest/user-guide/download/. The PennCNV algorithm incorporates multiple information sources, including the total signal intensity of the log R ratio (LRR), B allele frequency (BAF), and population frequency of the B allele (PFB)^54^. The LRR and BAF of each SNP for every sample were exported from Illumina GenomeStudio software. The PFB was generated based on the BAF of each SNP marker. Genomic waves were adjusted with the sheep GC model file, which was generated by calculating the GC content of 1-Mb genomic regions surrounding each marker (500 Kb on each side), after the program argument ‘gcmodel’ was used to adjust the results. According to previous research using high-density SNP chips to detect CNV in humans^55^,^56^, the CNV filter was based on three criteria: (1) the CNV must contain ten or more consecutive SNPs; (2) the length of the CNV must be at least 100 K; and (3) the CNV must be present in at least one animal.

Finally, following the method reported by Redon *et al*., CNVRs were obtained by merging CNVs with overlapping regions that were identified across all samples^7^.

### Experimental validation of CNVRs by quantitative PCR

Quantitative real-time PCR (qPCR) was used to validate the CNVRs that were detected in this study. First, qPCR was performed to validate 10 randomly selected CNVRs that were identified by PennCNV using genomic DNA of the same sheep as analysed by the Illumina chip. The GAPDH gene was chosen as a reference gene for all of the qPCR experiments^15^. The primers were designed using the Primer 3 webtool (http://frodo.wi.mit.edu/primer3/). All of the PCR primers were designed based on NCBI reference sequences (Supplementary File 1: Table S10). PCR reactions were prepared using the Power SYBR Green PCR reagent kit (Takara Bio). PCR was carried out in a total volume of 20 μL containing 1 μL of DNA (approximately 60 ng), forward primer and reverse primers (2 μL total), 10 μL of Master Mix (2×), and 15 μL of water. PCR was performed with the following cycling conditions: 5 min at 95 °C, followed by 40 cycles at 95 °C for 10 sec and 60 °C for 10 sec. All of the reactions were run in triplicate and included blank controls. The 2^−ΔΔCt^ method was used to calculate the copy number^57^,^58^,^59^, where Ct is the threshold cycle. The copy number of the target gene in the test sample was calculated as 2 × 2^−ΔΔCt^, where ΔΔCt was defined as in a previous report^60^. The corresponding equation was


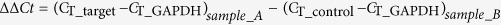


where C_t_ is the threshold cycle, sample A is the tested individual, and sample B is the control individual.

### Gene detection and functional analysis

Genes harboured in the inferred CNVRs were obtained from the Ensembl Genes 64 Database using BioMart software based on the *Ovis aries* (Oar_v3.1) gene sequence assembly. The Database for Annotation, Visualization and Integrated Discovery (DAVID) (http://david.abcc.ncifcrf.gov/) was used to perform the gene ontology (GO)^61^ enrichment analysis and Kyoto Encyclopedia of Gene and Genome (KEGG)^62^ pathway analysis. To better understand the functions of the genes within the detected CNVRs, the *Ovis aries* Ensembl gene IDs were converted into human orthologue Ensembl gene IDs using BioMart because the annotation of the sheep genome is limited.

## Additional Information

**How to cite this article**: Zhu, C. *et al*. Genome-wide detection of CNVs in Chinese indigenous sheep with different types of tails using ovine high-density 600K SNP arrays. *Sci. Rep.* **6**, 27822; doi: 10.1038/srep27822 (2016).

## Supplementary Material

Supplementary Information

## Figures and Tables

**Table 1:** Genome characteristics of copy number variation in three sheep breeds.

	Breed	Number	Total length (Mb)	Average length(Mb)	gain	loss	both	Percentage of chr. covered by CNVRs (%)
CNVs	Large-tailed Han sheep	2684	639.11	2.38	185	2498		–
	Altay sheep	2161	472.23	0.21	200	1963		–
	Tibetan sheep	356	63.82	0.17	92	264		–
CNVRs	Large-tailed Han sheep	371	71.35	1.92	44	326	1	2.75%
	Altay sheep	301	51.65	0.17	60	240	1	1.96%
	Tibetan sheep	66	10.56	0.16	10	54	2	0.41%

Note: CNVRs come from merging overlapping CNVs.

**Table 2:** Chromosome distribution of CNVRs in three sheep breeds.

Chr	No. of CNVRs	Length of CNVRs (bp)	>Length of chr (Mb)	Percentage (%)
1	34	4,773,633	275.61	1.73
2	42	7,208,651	248.99	2.89
3	58	10,216,936	224.28	4.56
4	12	1,645,575	119.26	1.38
5	24	3,620,608	107.9	3.36
6	20	4,426,168	117.03	3.78
7	14	2,555,658	100.08	2.55
8	6	1,369,453	90.7	1.50
9	8	1,427,087	94.73	1.50
10	19	4,109,934	86.45	4.75
11	39	5,295,426	62.25	8.51
12	19	2,816,084	79.1	3.56
13	15	2,165,290	83.08	2.61
14	23	3,787,011	62.72	6.04
15	24	3,945,723	80.92	4.88
16	11	2,111,046	71.72	2.94
17	24	4,304,308	72.29	5.95
18	17	2,828,822	68.6	4.12
19	14	2,106,692	60.46	3.48
20	14	2,026,056	51.18	3.96
21	9	1,087,096	50.07	2.17
22	7	1,069,148	50.83	2.10
23	9	1,572,457	62.33	2.52
24	17	2,639,607	42.03	6.28
25	8	1,363,031	45.37	3.00
26	3	570,889	44.08	1.29
Total	490	81,042,389	2452.06	3.30

**Table 3:** Genes associated with fat identified as located in CNVRs.

Breed	Chr	CNVR		Gene Symbol
		Start Position	End Position	
Large-tailed Han sheep	3	218,509,635	22,069,0926	*PPARA*
	3	30,394	4,358,207	*RXRA*
	3	19,105,792	19,909,972	*KLF11*
	6	115,199,394	115,766,842	*ADD1*
	11	49,764,393	50,272,168	*FASN*
	21	44,468,253	48,499,178	*PPP1CA*
	24	40,804,124	42,029,819	*PDGFA*
Altay sheep	3	30,394	2,523,294	*RXRA*
	11	49,876,846	50,231,458	*FASN*
	20	16,642,933	16,980,080	*PEX6*
	21	44,551,183	44,693,332	*PPP1CA*
	24	40,327,423	42,029,819	*PDGFA*
Tibetan sheep	3	1,408,687	1,845,573	*RXRA*

Note: Positions were retrieved from the sheep genome sequence assembly.

**Table 4:** CNVRs in common with previous studies in sheep.

	Platform	Sample	Number of CNVRs	Total length (Mb)	Concordant number with our study
This study	600 K SNP	110	490	81.04	–
Fontanesi *et al*.^15^	aCGH	11	135	10.5	11
Liu *et al*.^16^	50 K SNP	329	238	60.35	12
Ma *et al*.^17^	50 K SNP	160	111	13.75	8
