# Supplementary material for: Genome-wide detection of CNVs in Chinese indigenous sheep with different types of tails using ovine high-density 600K SNP arrays
Source: Sci Rep. 2016 Jun 10;6:27822. doi: 10.1038/srep27822 (PMC4901276; doi:10.1038/srep27822)
Supplement: Supplementary Information [file srep27822-s1.doc]

**Genome-wide detection of CNVs in Chinese indigenous sheep with different types of tails using Ovine high-density 600K SNP arrays**

Caiye Zhu^1,2,*^, Hongying Fan^1,3,*^, Zehu Yuan^1^, Shijin Hu^1^, Xiaomeng Ma^1^, Junli Xuan^3^, Hongwei Wang^4^, Li Zhang^1^, Caihong Wei^1^, Qin Zhang^2^, Fuping Zhao^1,5^, Lixin Du^1,5^

1 National Center for Molecular Genetics and Breeding of Animals, Institute of Animal Sciences, Chinese Academy of Agricultural Sciences, Beijing 100193, China;

2 College of Animal Science and Technology, China Agricultural University, Beijing 100193, China;

3 College of Animal Science and Technology, Gansu Agricultural University, Lanzhou, 730070, China;

4 Beijing Compass Biotechnology Co., Ltd，Beijing 100192, China

*These authors contributed equally to this work

^5^ Corresponding Authors:

Fuping Zhao (email: zhaofuping@caas.cn)

Lixin Du (email: lxdu@263.net)

Supplementary Figures

Figure S1 The distribution and status of detected CNVRs in Tibetan sheep.

Supplementary tables

**Table S1: the detailed features of CNVRs on autosomes identified in Large-tail Han sheep**

| **CNVR ID** | **Chr** | **Start(bp)** | **End(bp)** | **Length(bp)** | **No.SNP** | **Type** | **No.Samples** |
| --- | --- | --- | --- | --- | --- | --- | --- |
| 1 | 1 | 17218 | 1246467 | 147,366 | 262 | loss | 25 |
| 2 | 1 | 1542440 | 2339635 | 163,137 | 235 | loss | 7 |
| 3 | 1 | 2546815 | 3132186 | 169,077 | 169 | loss | 9 |
| 4 | 1 | 3269952 | 3643248 | 164,832 | 113 | loss | 4 |
| 5 | 1 | 7285572 | 7741112 | 169,225 | 125 | loss | 2 |
| 6 | 1 | 15069418 | 15178777 | 109,360 | 32 | loss | 2 |
| 7 | 1 | 20737305 | 20951770 | 111,398 | 58 | loss | 6 |
| 8 | 1 | 27721380 | 28025524 | 180,854 | 74 | loss | 5 |
| 9 | 1 | 28972525 | 29148388 | 108,967 | 55 | loss | 2 |
| 10 | 1 | 103744512 | 103888057 | 137,843 | 34 | loss | 4 |
| 11 | 1 | 104674851 | 104839975 | 107,876 | 47 | loss | 2 |
| 12 | 1 | 105354407 | 105655234 | 101,630 | 77 | loss | 4 |
| 13 | 1 | 121333493 | 121473502 | 140,010 | 67 | loss | 2 |
| 14 | 1 | 148587073 | 148765017 | 177,945 | 40 | gain | 5 |
| 15 | 1 | 189649273 | 189752265 | 102,993 | 36 | loss | 5 |
| 16 | 1 | 201011832 | 201214837 | 109,303 | 53 | loss | 5 |
| 17 | 1 | 260011540 | 260301261 | 128,799 | 92 | loss | 4 |
| 18 | 1 | 260933730 | 263519919 | 151,974 | 530 | loss | 17 |
| 19 | 1 | 263952533 | 264296166 | 103,286 | 129 | loss | 6 |
| 20 | 1 | 266476893 | 266619856 | 142,964 | 54 | loss | 2 |
| 21 | 1 | 271962142 | 272391202 | 429,061 | 107 | loss | 5 |
| 22 | 1 | 275198084 | 275579536 | 111,269 | 106 | loss | 7 |
| 23 | 2 | 17125 | 1973308 | 123,827 | 379 | loss | 23 |
| 24 | 2 | 9202943 | 9712161 | 205,042 | 162 | loss | 4 |
| 25 | 2 | 10266961 | 10376684 | 109,724 | 38 | loss | 2 |
| 26 | 2 | 27493574 | 27645627 | 143,472 | 41 | loss | 3 |
| 27 | 2 | 28185023 | 28409373 | 192,765 | 62 | loss | 4 |
| 28 | 2 | 43147217 | 43442188 | 105,670 | 73 | loss | 7 |
| 29 | 2 | 52513596 | 52630652 | 106,513 | 48 | loss | 2 |
| 30 | 2 | 110010145 | 110304256 | 294,112 | 82 | loss | 4 |
| 31 | 2 | 110683442 | 111254655 | 242,527 | 134 | loss | 6 |
| 32 | 2 | 113263009 | 113386994 | 123,986 | 22 | loss | 3 |
| 33 | 2 | 116674076 | 116859969 | 145,996 | 43 | loss | 7 |
| 34 | 2 | 117097356 | 117342715 | 217,230 | 60 | loss | 2 |
| 35 | 2 | 122385838 | 122869959 | 484,122 | 84 | gain | 3 |
| 36 | 2 | 132818788 | 132940252 | 121,465 | 30 | loss | 3 |
| 37 | 2 | 184750247 | 185025475 | 260,260 | 55 | loss | 2 |
| 38 | 2 | 194252627 | 194563518 | 310,892 | 47 | gain | 2 |
| 39 | 2 | 218798138 | 219144586 | 165,691 | 75 | loss | 5 |
| 40 | 2 | 220088311 | 220539843 | 144,556 | 146 | loss | 12 |
| 41 | 2 | 232234842 | 232472561 | 190,675 | 63 | loss | 2 |
| 42 | 2 | 233076544 | 233252525 | 110,140 | 50 | loss | 5 |
| 43 | 2 | 234745397 | 235142194 | 396,798 | 115 | loss | 2 |
| 44 | 2 | 238515681 | 238871634 | 355,954 | 86 | loss | 2 |
| 45 | 2 | 240735937 | 240992124 | 250,169 | 62 | loss | 3 |
| 46 | 2 | 242723012 | 243204353 | 139,832 | 161 | loss | 8 |
| 47 | 2 | 243353510 | 243603367 | 249,858 | 60 | loss | 2 |
| 48 | 2 | 243703321 | 243878590 | 161,409 | 53 | loss | 3 |
| 49 | 2 | 246009958 | 246428847 | 185,943 | 72 | loss | 7 |
| 50 | 2 | 246541978 | 248981782 | 134,391 | 570 | loss | 30 |
| 51 | 3 | 30394 | 4358207 | 368,570 | 973 | loss | 35 |
| 52 | 3 | 4969959 | 5325867 | 167,231 | 92 | loss | 4 |
| 53 | 3 | 6753855 | 6884511 | 118,191 | 31 | loss | 5 |
| 54 | 3 | 7516773 | 7719320 | 154,575 | 50 | loss | 3 |
| 55 | 3 | 8525964 | 8668862 | 142,899 | 40 | loss | 2 |
| 56 | 3 | 8834343 | 8992087 | 157,745 | 43 | loss | 2 |
| 57 | 3 | 13532931 | 13661207 | 108,525 | 38 | loss | 2 |
| 58 | 3 | 13959873 | 14205181 | 245,309 | 54 | loss | 4 |
| 59 | 3 | 17921712 | 18106593 | 100,643 | 50 | loss | 4 |
| 60 | 3 | 18417040 | 18673107 | 241,647 | 81 | loss | 2 |
| 61 | 3 | 19105792 | 19909972 | 804,181 | 197 | loss | 2 |
| 62 | 3 | 57360189 | 57560618 | 126,063 | 44 | loss | 4 |
| 63 | 3 | 62001931 | 62275376 | 273,446 | 93 | loss | 2 |
| 64 | 3 | 88753699 | 88890614 | 136,916 | 27 | gain | 2 |
| 65 | 3 | 96153092 | 96351441 | 104,201 | 73 | loss | 8 |
| 66 | 3 | 97091525 | 97513405 | 421,881 | 121 | loss | 2 |
| 67 | 3 | 102220378 | 102461636 | 241,259 | 65 | loss | 2 |
| 68 | 3 | 102630095 | 102782494 | 152,400 | 47 | loss | 8 |
| 69 | 3 | 103254925 | 104402177 | 112,746 | 305 | loss | 10 |
| 70 | 3 | 120401806 | 120822876 | 421,071 | 89 | gain | 2 |
| 71 | 3 | 132193852 | 132444146 | 149,231 | 55 | loss | 5 |
| 72 | 3 | 133957365 | 134304682 | 163,705 | 98 | loss | 4 |
| 73 | 3 | 136421489 | 136533303 | 102,196 | 34 | loss | 2 |
| 74 | 3 | 136934261 | 137055281 | 106,821 | 29 | loss | 3 |
| 75 | 3 | 137512720 | 137614332 | 101,613 | 24 | loss | 4 |
| 76 | 3 | 138464202 | 138664939 | 114,865 | 67 | loss | 4 |
| 77 | 3 | 158125037 | 158266189 | 141,153 | 32 | gain | 2 |
| 78 | 3 | 161485024 | 161915835 | 142,685 | 113 | loss | 6 |
| 79 | 3 | 162078846 | 162355920 | 277,075 | 71 | loss | 3 |
| 80 | 3 | 164004285 | 164300378 | 110,824 | 43 | gain | 7 |
| 81 | 3 | 178354420 | 178814735 | 407,028 | 132 | loss | 4 |
| 82 | 3 | 179528439 | 179844384 | 315,946 | 88 | loss | 2 |
| 83 | 3 | 180518729 | 180841024 | 322,296 | 72 | loss | 2 |
| 84 | 3 | 204182428 | 204310421 | 118,182 | 31 | gain | 2 |
| 85 | 3 | 207457282 | 208110083 | 109,960 | 185 | loss | 4 |
| 86 | 3 | 210892488 | 211005685 | 102,146 | 46 | loss | 2 |
| 87 | 3 | 212055171 | 212628102 | 572,932 | 152 | loss | 2 |
| 88 | 3 | 212686337 | 212937950 | 162,122 | 59 | loss | 6 |
| 89 | 3 | 213001360 | 214138526 | 265,584 | 182 | loss | 23 |
| 90 | 3 | 214651731 | 214788769 | 137,039 | 39 | loss | 2 |
| 91 | 3 | 216884648 | 216999700 | 101,913 | 34 | loss | 3 |
| 92 | 3 | 217226491 | 218351678 | 113,673 | 330 | loss | 4 |
| 93 | 3 | 218509635 | 220690926 | 117,103 | 294 | loss | 11 |
| 94 | 3 | 220724137 | 221525942 | 141,112 | 196 | loss | 10 |
| 95 | 3 | 222525469 | 222861323 | 136,366 | 59 | loss | 6 |
| 96 | 3 | 223279489 | 224278204 | 179,791 | 182 | loss | 23 |
| 97 | 4 | 65211323 | 65327602 | 116,280 | 32 | loss | 2 |
| 98 | 4 | 68793125 | 68942168 | 105,397 | 31 | loss | 24 |
| 99 | 4 | 76523880 | 76699046 | 107,584 | 53 | loss | 2 |
| 100 | 4 | 77145243 | 77261687 | 116,445 | 43 | loss | 2 |
| 101 | 4 | 92817945 | 92977735 | 104,872 | 60 | loss | 9 |
| 102 | 4 | 98785415 | 98943045 | 157,631 | 52 | loss | 2 |
| 103 | 4 | 111666749 | 112128525 | 137,061 | 148 | loss | 3 |
| 104 | 4 | 112913781 | 113244910 | 102,559 | 80 | loss | 19 |
| 105 | 4 | 116217849 | 116452157 | 208,127 | 64 | loss | 7 |
| 106 | 4 | 116571716 | 119102454 | 528,017 | 491 | loss | 14 |
| 107 | 5 | 1576810 | 1880404 | 212,614 | 85 | loss | 2 |
| 108 | 5 | 2574241 | 2714621 | 104,830 | 46 | loss | 2 |
| 109 | 5 | 3433954 | 4823218 | 217,000 | 411 | loss | 4 |
| 110 | 5 | 5526844 | 5638078 | 111,235 | 52 | loss | 2 |
| 111 | 5 | 9302707 | 10003364 | 224,731 | 222 | loss | 5 |
| 112 | 5 | 10393615 | 10938908 | 498,610 | 141 | loss | 2 |
| 113 | 5 | 12337169 | 13599186 | 1,262,018 | 308 | loss | 2 |
| 114 | 5 | 14261191 | 14460794 | 199,604 | 58 | loss | 2 |
| 115 | 5 | 15607771 | 15811834 | 204,064 | 51 | loss | 2 |
| 116 | 5 | 15973003 | 16348376 | 100,803 | 84 | loss | 12 |
| 117 | 5 | 16681548 | 19154728 | 125,728 | 584 | loss | 16 |
| 118 | 5 | 19942912 | 20101166 | 114,527 | 41 | loss | 5 |
| 119 | 5 | 35478809 | 35653159 | 117,893 | 69 | loss | 3 |
| 120 | 5 | 35722404 | 35829833 | 107,430 | 27 | loss | 2 |
| 121 | 5 | 38973944 | 39095642 | 109,002 | 17 | gain | 2 |
| 122 | 5 | 40429310 | 41558353 | 284,787 | 236 | loss | 18 |
| 123 | 5 | 48248624 | 48448480 | 175,748 | 47 | loss | 2 |
| 124 | 5 | 59804966 | 59954532 | 149,567 | 31 | loss | 2 |
| 125 | 5 | 82475727 | 83213540 | 166,191 | 108 | gain | 5 |
| 126 | 6 | 31930104 | 32270757 | 340,654 | 74 | gain | 2 |
| 127 | 6 | 33930366 | 34308545 | 378,180 | 78 | gain | 2 |
| 128 | 6 | 37325169 | 37451479 | 126,311 | 28 | gain | 5 |
| 129 | 6 | 51471221 | 51892837 | 421,617 | 82 | gain | 3 |
| 130 | 6 | 64665847 | 64775497 | 109,651 | 20 | gain | 2 |
| 131 | 6 | 74004726 | 74138753 | 134,028 | 26 | gain | 2 |
| 132 | 6 | 75285216 | 75508227 | 223,012 | 39 | gain | 2 |
| 133 | 6 | 78155315 | 80029344 | 224,925 | 317 | gain | 4 |
| 134 | 6 | 85164829 | 85309224 | 131,708 | 38 | gain | 4 |
| 135 | 6 | 102185688 | 102889568 | 703,881 | 232 | loss | 3 |
| 136 | 6 | 112634642 | 112789113 | 138,884 | 45 | loss | 2 |
| 137 | 6 | 113781723 | 113957987 | 105,209 | 29 | loss | 4 |
| 138 | 6 | 114279024 | 114466560 | 102,293 | 55 | loss | 2 |
| 139 | 6 | 114711090 | 115094734 | 121,755 | 86 | loss | 18 |
| 140 | 6 | 115199394 | 115766842 | 174,136 | 82 | loss | 13 |
| 141 | 6 | 115908289 | 117025755 | 182,940 | 154 | loss | 32 |
| 142 | 7 | 13262576 | 13426522 | 108,904 | 40 | loss | 4 |
| 143 | 7 | 20098704 | 20730415 | 389,448 | 161 | loss | 3 |
| 144 | 7 | 21002815 | 21240846 | 192,912 | 69 | loss | 3 |
| 145 | 7 | 22342026 | 22581939 | 239,914 | 39 | gain | 3 |
| 146 | 7 | 22597761 | 22705659 | 107,899 | 14 | gain | 2 |
| 147 | 7 | 32875841 | 33109128 | 100,968 | 51 | loss | 9 |
| 148 | 7 | 33327726 | 33506754 | 179,029 | 61 | loss | 2 |
| 149 | 7 | 82705699 | 82924327 | 154,139 | 68 | loss | 2 |
| 150 | 7 | 84595003 | 84798923 | 187,134 | 62 | loss | 4 |
| 151 | 7 | 84982372 | 85480686 | 498,315 | 128 | loss | 2 |
| 152 | 7 | 96751386 | 97144392 | 377,148 | 107 | loss | 2 |
| 153 | 7 | 97554990 | 98147160 | 592,171 | 148 | loss | 3 |
| 154 | 7 | 98496118 | 99676548 | 178,772 | 305 | loss | 10 |
| 155 | 8 | 2222170 | 2437064 | 173,186 | 32 | gain | 17 |
| 156 | 8 | 83411549 | 83547691 | 136,143 | 43 | loss | 2 |
| 157 | 8 | 88470881 | 90492859 | 236,643 | 413 | loss | 24 |
| 158 | 9 | 13193845 | 16596810 | 133,174 | 900 | loss | 34 |
| 159 | 9 | 63086070 | 63406906 | 320,837 | 69 | gain | 2 |
| 160 | 9 | 77377746 | 77563420 | 185,675 | 20 | gain | 2 |
| 161 | 9 | 93876067 | 93995655 | 116,882 | 32 | loss | 4 |
| 162 | 10 | 7362107 | 8173096 | 597,914 | 122 | gain | 4 |
| 163 | 10 | 21498197 | 21720606 | 222,410 | 78 | loss | 2 |
| 164 | 10 | 32388248 | 32502028 | 113,781 | 31 | loss | 2 |
| 165 | 10 | 34408109 | 34870291 | 164,373 | 124 | loss | 3 |
| 166 | 10 | 35390498 | 35728094 | 238,626 | 70 | loss | 2 |
| 167 | 10 | 35838530 | 36338933 | 500,404 | 109 | loss | 3 |
| 168 | 10 | 36847123 | 36959483 | 112,361 | 34 | loss | 2 |
| 169 | 10 | 38266408 | 38522127 | 136,706 | 41 | gain | 2 |
| 170 | 10 | 43431360 | 43559357 | 127,998 | 22 | gain | 4 |
| 171 | 10 | 70548274 | 70883623 | 133,372 | 61 | gain | 9 |
| 172 | 10 | 70952059 | 71162168 | 188,580 | 28 | gain-loss | 4 |
| 173 | 10 | 71679652 | 71797990 | 118,339 | 26 | gain | 2 |
| 174 | 10 | 84575318 | 84977613 | 192,074 | 61 | loss | 4 |
| 175 | 10 | 85403492 | 85566962 | 129,509 | 36 | loss | 6 |
| 176 | 10 | 85689187 | 86439042 | 203,221 | 122 | loss | 14 |
| 177 | 11 | 11167913 | 11287843 | 119,931 | 37 | loss | 2 |
| 178 | 11 | 18080459 | 18238141 | 144,707 | 49 | loss | 2 |
| 179 | 11 | 19474851 | 20175974 | 701,124 | 205 | loss | 3 |
| 180 | 11 | 21216407 | 21716773 | 280,171 | 142 | loss | 4 |
| 181 | 11 | 21868581 | 22113776 | 245,196 | 73 | loss | 2 |
| 182 | 11 | 22218565 | 22472972 | 102,499 | 83 | loss | 5 |
| 183 | 11 | 22566832 | 22728568 | 161,737 | 35 | loss | 3 |
| 184 | 11 | 23195819 | 23505479 | 309,661 | 87 | loss | 2 |
| 185 | 11 | 24545528 | 24729579 | 155,724 | 63 | loss | 6 |
| 186 | 11 | 26550012 | 27498215 | 134,106 | 142 | loss | 12 |
| 187 | 11 | 27816888 | 28062254 | 133,633 | 68 | loss | 5 |
| 188 | 11 | 33607756 | 33811826 | 204,071 | 48 | loss | 2 |
| 189 | 11 | 33868688 | 34597636 | 142,472 | 101 | loss | 17 |
| 190 | 11 | 41673116 | 41796063 | 122,948 | 39 | loss | 2 |
| 191 | 11 | 47175399 | 47305094 | 110,617 | 41 | loss | 3 |
| 192 | 11 | 48924471 | 49038684 | 110,530 | 37 | loss | 2 |
| 193 | 11 | 49498051 | 49688184 | 190,134 | 55 | loss | 2 |
| 194 | 11 | 49764393 | 50272168 | 118,394 | 92 | loss | 8 |
| 195 | 11 | 50464670 | 50814716 | 111,277 | 98 | loss | 4 |
| 196 | 11 | 51406283 | 51570512 | 147,370 | 60 | loss | 4 |
| 197 | 11 | 52226107 | 52550458 | 116,246 | 120 | loss | 3 |
| 198 | 11 | 52906832 | 53074284 | 167,453 | 58 | loss | 2 |
| 199 | 11 | 54258558 | 54467069 | 195,980 | 64 | loss | 4 |
| 200 | 11 | 54529615 | 55063612 | 106,284 | 192 | loss | 18 |
| 201 | 11 | 55223012 | 55673721 | 132,876 | 102 | loss | 14 |
| 202 | 11 | 55905371 | 56372885 | 155,665 | 145 | loss | 4 |
| 203 | 11 | 56630543 | 56823143 | 101,391 | 35 | loss | 2 |
| 204 | 11 | 56897973 | 57068226 | 170,254 | 60 | loss | 2 |
| 205 | 11 | 60585053 | 60774789 | 106,140 | 60 | loss | 2 |
| 206 | 11 | 60981089 | 61284563 | 170,381 | 75 | loss | 6 |
| 207 | 11 | 61761425 | 62043545 | 221,176 | 58 | loss | 4 |
| 208 | 12 | 1190747 | 1315546 | 124,800 | 48 | loss | 2 |
| 209 | 12 | 2030972 | 2156494 | 119,174 | 44 | loss | 2 |
| 210 | 12 | 3500281 | 3648368 | 128,606 | 50 | loss | 6 |
| 211 | 12 | 25010450 | 25204961 | 171,298 | 78 | loss | 2 |
| 212 | 12 | 35743933 | 35845891 | 101,959 | 23 | gain | 5 |
| 213 | 12 | 40012073 | 40172294 | 160,222 | 52 | loss | 4 |
| 214 | 12 | 40946885 | 41116411 | 106,872 | 44 | loss | 7 |
| 215 | 12 | 41763886 | 41972201 | 186,185 | 53 | loss | 5 |
| 216 | 12 | 42211434 | 42486394 | 165,607 | 68 | loss | 5 |
| 217 | 12 | 43774586 | 44052246 | 249,499 | 75 | loss | 3 |
| 218 | 12 | 44888051 | 45585048 | 127,456 | 200 | loss | 15 |
| 219 | 12 | 47457266 | 48091242 | 122,314 | 179 | loss | 12 |
| 220 | 12 | 48125481 | 48915546 | 103,450 | 168 | loss | 16 |
| 221 | 12 | 49002167 | 49901432 | 117,445 | 118 | loss | 14 |
| 222 | 12 | 61964043 | 62127531 | 163,489 | 40 | gain | 2 |
| 223 | 12 | 75166383 | 75290670 | 105,060 | 30 | loss | 2 |
| 224 | 12 | 77484498 | 77975354 | 206,222 | 129 | loss | 11 |
| 225 | 12 | 78244161 | 79070188 | 315,528 | 145 | loss | 23 |
| 226 | 13 | 45193041 | 45758178 | 101,406 | 187 | loss | 2 |
| 227 | 13 | 52996440 | 54985635 | 132,345 | 520 | loss | 29 |
| 228 | 13 | 59638548 | 59764923 | 126,376 | 31 | loss | 2 |
| 229 | 13 | 60370980 | 60533120 | 125,551 | 37 | loss | 3 |
| 230 | 13 | 74144539 | 74321307 | 176,769 | 47 | loss | 2 |
| 231 | 13 | 78374859 | 78475773 | 100,915 | 30 | loss | 2 |
| 232 | 13 | 78742887 | 78852781 | 104,612 | 30 | loss | 3 |
| 233 | 13 | 81267177 | 81406516 | 102,063 | 41 | loss | 2 |
| 234 | 13 | 82653401 | 83022168 | 368,768 | 90 | loss | 2 |
| 235 | 14 | 1203994 | 1320060 | 116,067 | 33 | loss | 2 |
| 236 | 14 | 1821148 | 1989592 | 165,008 | 44 | loss | 4 |
| 237 | 14 | 7416410 | 7874805 | 414,649 | 118 | loss | 5 |
| 238 | 14 | 10773826 | 11226484 | 144,364 | 110 | loss | 5 |
| 239 | 14 | 12399710 | 14324839 | 151,939 | 403 | loss | 17 |
| 240 | 14 | 33892398 | 34285629 | 139,965 | 67 | loss | 12 |
| 241 | 14 | 34384667 | 34805062 | 126,097 | 105 | loss | 5 |
| 242 | 14 | 42888243 | 43590748 | 667,139 | 179 | loss | 4 |
| 243 | 14 | 44840080 | 44947670 | 107,591 | 36 | loss | 2 |
| 244 | 14 | 45587237 | 45893975 | 306,739 | 116 | loss | 2 |
| 245 | 14 | 47165085 | 47672055 | 506,971 | 123 | loss | 2 |
| 246 | 14 | 48002289 | 48388672 | 354,289 | 105 | loss | 2 |
| 247 | 14 | 48738235 | 49223740 | 165,309 | 137 | loss | 9 |
| 248 | 14 | 49934777 | 50507077 | 130,886 | 134 | loss | 8 |
| 249 | 14 | 50888672 | 51076943 | 149,960 | 63 | loss | 2 |
| 250 | 14 | 51694078 | 52051702 | 357,625 | 84 | loss | 2 |
| 251 | 14 | 52369849 | 52957964 | 148,357 | 152 | loss | 6 |
| 252 | 14 | 54891002 | 55242725 | 109,921 | 99 | loss | 5 |
| 253 | 14 | 59107917 | 59406372 | 129,449 | 55 | loss | 9 |
| 254 | 14 | 59460199 | 59610579 | 133,806 | 66 | loss | 2 |
| 255 | 14 | 62426919 | 62712806 | 237,574 | 70 | loss | 8 |
| 256 | 15 | 2260784 | 2937065 | 475,198 | 120 | gain | 2 |
| 257 | 15 | 10274423 | 11203694 | 319,159 | 129 | gain | 11 |
| 258 | 15 | 11868955 | 12101107 | 201,993 | 42 | gain | 3 |
| 259 | 15 | 27361124 | 27563048 | 195,168 | 72 | loss | 2 |
| 260 | 15 | 43725680 | 43898728 | 173,049 | 51 | loss | 2 |
| 261 | 15 | 45601527 | 45787694 | 149,586 | 80 | gain | 19 |
| 262 | 15 | 45814480 | 46061865 | 114,607 | 93 | loss | 5 |
| 263 | 15 | 50342943 | 50486417 | 143,475 | 44 | loss | 2 |
| 264 | 15 | 52789409 | 52949888 | 160,480 | 48 | loss | 3 |
| 265 | 15 | 53733768 | 53839596 | 105,829 | 24 | loss | 2 |
| 266 | 15 | 54203565 | 54349173 | 145,609 | 54 | loss | 2 |
| 267 | 15 | 54576023 | 54755666 | 175,122 | 60 | loss | 5 |
| 268 | 15 | 74068333 | 74199604 | 100,332 | 38 | loss | 4 |
| 269 | 15 | 74475270 | 74645248 | 169,979 | 49 | loss | 2 |
| 270 | 15 | 75297169 | 75625948 | 328,780 | 72 | loss | 2 |
| 271 | 15 | 77672081 | 78220052 | 547,972 | 155 | loss | 3 |
| 272 | 15 | 80410579 | 80829855 | 165,014 | 86 | loss | 14 |
| 273 | 16 | 473598 | 797065 | 323,468 | 96 | loss | 2 |
| 274 | 16 | 27372078 | 27690818 | 318,741 | 74 | gain | 2 |
| 275 | 16 | 47189647 | 48069203 | 149,589 | 168 | gain | 8 |
| 276 | 16 | 52755375 | 53017137 | 261,763 | 56 | gain | 2 |
| 277 | 16 | 69943027 | 70206922 | 152,680 | 92 | loss | 4 |
| 278 | 16 | 70469116 | 71016525 | 126,039 | 76 | loss | 16 |
| 279 | 16 | 71198072 | 71715748 | 100,731 | 71 | loss | 24 |
| 280 | 17 | 21474397 | 21931716 | 243,055 | 82 | gain | 3 |
| 281 | 17 | 23381788 | 23496203 | 114,416 | 28 | gain | 2 |
| 282 | 17 | 26722671 | 26953030 | 101,391 | 38 | gain | 9 |
| 283 | 17 | 44024856 | 44894126 | 107,037 | 249 | loss | 12 |
| 284 | 17 | 50809428 | 51095849 | 137,973 | 52 | loss | 10 |
| 285 | 17 | 52107896 | 52286741 | 169,816 | 34 | loss | 3 |
| 286 | 17 | 60237606 | 60404477 | 155,837 | 69 | loss | 2 |
| 287 | 17 | 62096410 | 62247317 | 150,908 | 43 | loss | 7 |
| 288 | 17 | 63031657 | 63257791 | 226,135 | 52 | loss | 2 |
| 289 | 17 | 68357082 | 68470428 | 113,347 | 37 | loss | 2 |
| 290 | 17 | 68806525 | 69560911 | 754,387 | 227 | loss | 4 |
| 291 | 17 | 69710844 | 69882461 | 163,588 | 71 | loss | 3 |
| 292 | 17 | 70443207 | 72279291 | 141,001 | 391 | loss | 31 |
| 293 | 18 | 1802043 | 2023593 | 221,551 | 64 | loss | 2 |
| 294 | 18 | 12207155 | 12653998 | 439,761 | 106 | gain | 3 |
| 295 | 18 | 19488042 | 19599474 | 111,433 | 39 | loss | 2 |
| 296 | 18 | 19904707 | 20085240 | 180,534 | 46 | loss | 4 |
| 297 | 18 | 29349010 | 29565671 | 216,662 | 57 | loss | 2 |
| 298 | 18 | 31786788 | 32136593 | 320,778 | 118 | loss | 4 |
| 299 | 18 | 32365541 | 32487822 | 122,282 | 25 | loss | 4 |
| 300 | 18 | 32673708 | 32846550 | 120,600 | 42 | loss | 2 |
| 301 | 18 | 51000939 | 51115352 | 114,414 | 18 | gain | 2 |
| 302 | 18 | 63834719 | 63946456 | 111,738 | 32 | loss | 2 |
| 303 | 18 | 64623786 | 66645322 | 159,677 | 446 | loss | 8 |
| 304 | 18 | 66899088 | 67026870 | 127,783 | 41 | loss | 2 |
| 305 | 18 | 67106299 | 67737127 | 151,276 | 99 | loss | 12 |
| 306 | 18 | 67771953 | 68601777 | 125,502 | 134 | loss | 19 |
| 307 | 19 | 11368559 | 11524505 | 155,947 | 49 | loss | 2 |
| 308 | 19 | 47122415 | 47255723 | 133,309 | 32 | loss | 2 |
| 309 | 19 | 48324183 | 48976603 | 102,910 | 194 | loss | 7 |
| 310 | 19 | 49651397 | 50168046 | 241,729 | 112 | loss | 12 |
| 311 | 19 | 50282691 | 50612222 | 110,856 | 104 | loss | 7 |
| 312 | 19 | 50792800 | 50965294 | 149,415 | 55 | loss | 4 |
| 313 | 19 | 51141275 | 51403316 | 103,720 | 63 | loss | 7 |
| 314 | 19 | 52428671 | 52580066 | 131,243 | 45 | loss | 16 |
| 315 | 19 | 54197433 | 54454621 | 193,058 | 77 | loss | 6 |
| 316 | 19 | 55980216 | 56126158 | 132,629 | 36 | loss | 5 |
| 317 | 19 | 57232845 | 57395959 | 151,346 | 41 | loss | 2 |
| 318 | 19 | 57748802 | 57927983 | 179,182 | 54 | loss | 2 |
| 319 | 19 | 58541953 | 58849114 | 217,289 | 63 | loss | 4 |
| 320 | 19 | 58926990 | 59367421 | 154,304 | 88 | loss | 9 |
| 321 | 19 | 59515354 | 60446408 | 217,388 | 189 | loss | 22 |
| 322 | 20 | 8247650 | 8446449 | 106,830 | 45 | loss | 9 |
| 323 | 20 | 8593363 | 8722533 | 118,363 | 28 | loss | 2 |
| 324 | 20 | 9396217 | 9567234 | 171,018 | 47 | loss | 2 |
| 325 | 20 | 10937086 | 11080463 | 118,181 | 42 | loss | 2 |
| 326 | 20 | 15415171 | 15566192 | 140,896 | 37 | loss | 2 |
| 327 | 20 | 16642933 | 16980080 | 123,880 | 70 | loss | 10 |
| 328 | 20 | 49322760 | 49449119 | 100,055 | 28 | loss | 2 |
| 329 | 20 | 49913946 | 51159416 | 120,805 | 90 | loss | 15 |
| 330 | 21 | 35682186 | 35796243 | 114,058 | 36 | loss | 2 |
| 331 | 21 | 39533129 | 39638962 | 100,310 | 31 | loss | 6 |
| 332 | 21 | 41424501 | 41989188 | 240,273 | 142 | loss | 4 |
| 333 | 21 | 42150108 | 42735679 | 242,380 | 156 | loss | 4 |
| 334 | 21 | 43023084 | 43403450 | 111,669 | 102 | loss | 11 |
| 335 | 21 | 44149257 | 44350445 | 171,972 | 48 | loss | 2 |
| 336 | 21 | 44468253 | 48499178 | 117,034 | 538 | loss | 19 |
| 337 | 21 | 48579849 | 48831283 | 150,709 | 38 | loss | 9 |
| 338 | 21 | 48891828 | 49104226 | 184,041 | 38 | loss | 4 |
| 339 | 21 | 49138922 | 49995820 | 300,650 | 86 | loss | 27 |
| 340 | 22 | 1849424 | 2052293 | 106,822 | 37 | gain | 2 |
| 341 | 22 | 20954454 | 21243674 | 284,501 | 79 | loss | 4 |
| 342 | 22 | 23103977 | 23429477 | 194,623 | 92 | loss | 5 |
| 343 | 22 | 42958926 | 43189643 | 130,915 | 57 | loss | 7 |
| 344 | 22 | 47990537 | 48203094 | 212,558 | 55 | loss | 3 |
| 345 | 22 | 49006159 | 49122945 | 116,787 | 43 | loss | 2 |
| 346 | 22 | 49744172 | 50828450 | 484,741 | 135 | loss | 22 |
| 347 | 23 | 523457 | 848215 | 267,826 | 65 | loss | 6 |
| 348 | 23 | 996677 | 1228581 | 102,097 | 64 | loss | 2 |
| 349 | 23 | 46291143 | 46424743 | 127,749 | 47 | loss | 2 |
| 350 | 23 | 60207975 | 60752989 | 545,015 | 170 | loss | 2 |
| 351 | 23 | 60939369 | 61873507 | 212,770 | 219 | loss | 11 |
| 352 | 24 | 19967 | 1380176 | 118,519 | 382 | loss | 25 |
| 353 | 24 | 1454321 | 1821735 | 123,520 | 82 | loss | 18 |
| 354 | 24 | 1927823 | 2508661 | 143,684 | 136 | loss | 14 |
| 355 | 24 | 2938579 | 3071910 | 133,332 | 68 | loss | 2 |
| 356 | 24 | 3432193 | 3858265 | 183,368 | 104 | loss | 10 |
| 357 | 24 | 3917090 | 4128679 | 107,159 | 84 | loss | 5 |
| 358 | 24 | 7634259 | 7780633 | 146,375 | 38 | loss | 2 |
| 359 | 24 | 24784697 | 25055998 | 271,302 | 76 | loss | 3 |
| 360 | 24 | 26369853 | 26565757 | 195,905 | 66 | loss | 2 |
| 361 | 24 | 26875194 | 27237559 | 362,366 | 101 | loss | 2 |
| 362 | 24 | 33638609 | 33751167 | 112,559 | 37 | loss | 3 |
| 363 | 24 | 35017019 | 35178906 | 161,888 | 40 | loss | 2 |
| 364 | 24 | 35748028 | 35872661 | 124,634 | 25 | loss | 3 |
| 365 | 24 | 37177842 | 38345534 | 121,335 | 331 | loss | 15 |
| 366 | 24 | 38984006 | 39170476 | 104,360 | 60 | loss | 6 |
| 367 | 24 | 40461330 | 40688193 | 101,415 | 50 | loss | 6 |
| 368 | 24 | 40804124 | 42029819 | 285,695 | 191 | loss | 31 |
| 369 | 25 | 2258259 | 2410224 | 151,966 | 35 | loss | 2 |
| 370 | 25 | 27187968 | 27832372 | 104,738 | 212 | loss | 4 |
| 371 | 26 | 1054696 | 1299859 | 100,835 | 75 | loss | 3 |

Table S2: the detailed features of CNVRs on autosomes identified in Altay sheep

| **CNVR ID** | **Chr** | **Start(bp)** | **End(bp)** | **Length(bp)** | **No.SNP** | **Type** | **No.Samples** |
| --- | --- | --- | --- | --- | --- | --- | --- |
| 1 | 1 | 17218 | 1282340 | 147,366 | 268 | loss | 30 |
| 2 | 1 | 1765272 | 1884072 | 118,801 | 33 | loss | 2 |
| 3 | 1 | 1915945 | 2288963 | 215,283 | 71 | loss | 6 |
| 4 | 1 | 2887631 | 2995306 | 107,676 | 42 | loss | 2 |
| 5 | 1 | 20763894 | 20879819 | 103,554 | 34 | loss | 2 |
| 6 | 1 | 27757627 | 28025524 | 155,120 | 64 | loss | 2 |
| 7 | 1 | 48764405 | 49055973 | 195,416 | 48 | gain | 4 |
| 8 | 1 | 79973027 | 80101789 | 120,486 | 21 | gain | 2 |
| 9 | 1 | 105354407 | 105481856 | 127,450 | 39 | loss | 2 |
| 10 | 1 | 110407863 | 110530871 | 123,009 | 29 | gain | 2 |
| 11 | 1 | 148587073 | 148866436 | 119,955 | 60 | gain | 4 |
| 12 | 1 | 189649273 | 189749385 | 100,113 | 35 | loss | 2 |
| 13 | 1 | 201011832 | 201216289 | 204,458 | 55 | loss | 2 |
| 14 | 1 | 252954232 | 253096396 | 142,165 | 40 | loss | 2 |
| 15 | 1 | 261937690 | 262134414 | 196,725 | 58 | loss | 2 |
| 16 | 1 | 262315705 | 262571682 | 121,959 | 79 | loss | 4 |
| 17 | 1 | 263024873 | 263410572 | 125,604 | 85 | loss | 10 |
| 18 | 1 | 263608231 | 263782431 | 167,214 | 48 | loss | 2 |
| 19 | 1 | 263869952 | 264134753 | 132,424 | 61 | loss | 5 |
| 20 | 1 | 270483710 | 270598507 | 114,798 | 35 | loss | 2 |
| 21 | 1 | 275307455 | 275563251 | 230,623 | 68 | loss | 6 |
| 22 | 2 | 17125 | 1094208 | 107,921 | 191 | loss | 23 |
| 23 | 2 | 1635335 | 1963517 | 138,861 | 84 | loss | 5 |
| 24 | 2 | 2874473 | 3218221 | 343,749 | 120 | loss | 3 |
| 25 | 2 | 43132271 | 43450508 | 126,433 | 78 | loss | 4 |
| 26 | 2 | 110810355 | 111119556 | 256,769 | 73 | loss | 4 |
| 27 | 2 | 116703428 | 116849423 | 145,996 | 38 | loss | 3 |
| 28 | 2 | 117097356 | 117342715 | 224,165 | 60 | loss | 3 |
| 29 | 2 | 184927029 | 185211174 | 190,986 | 55 | loss | 2 |
| 30 | 2 | 218889476 | 219162459 | 137,333 | 58 | loss | 4 |
| 31 | 2 | 220317382 | 220539843 | 222,462 | 71 | loss | 2 |
| 32 | 2 | 232254769 | 232472561 | 217,793 | 60 | loss | 2 |
| 33 | 2 | 233121670 | 233286379 | 101,227 | 30 | loss | 7 |
| 34 | 2 | 234304860 | 234447170 | 142,311 | 39 | loss | 3 |
| 35 | 2 | 238565587 | 238810442 | 244,856 | 59 | loss | 2 |
| 36 | 2 | 240735937 | 240954466 | 218,530 | 51 | loss | 3 |
| 37 | 2 | 244944729 | 245080590 | 116,832 | 38 | loss | 2 |
| 38 | 2 | 245578646 | 245683288 | 104,643 | 28 | loss | 2 |
| 39 | 2 | 246527777 | 246659687 | 103,573 | 45 | loss | 2 |
| 40 | 2 | 246950972 | 247056898 | 105,927 | 37 | loss | 2 |
| 41 | 2 | 247796045 | 247938541 | 142,497 | 39 | loss | 2 |
| 42 | 2 | 247969339 | 248578600 | 134,016 | 142 | loss | 9 |
| 43 | 2 | 248702542 | 248981782 | 116,361 | 65 | loss | 13 |
| 44 | 3 | 30394 | 2523294 | 105,161 | 201 | loss | 34 |
| 45 | 3 | 2650160 | 3853617 | 145,751 | 205 | loss | 23 |
| 46 | 3 | 4150738 | 4275414 | 113,295 | 36 | loss | 2 |
| 47 | 3 | 5011133 | 5289567 | 278,435 | 76 | loss | 2 |
| 48 | 3 | 5542371 | 5728084 | 106,430 | 77 | loss | 3 |
| 49 | 3 | 6753855 | 6884511 | 130,657 | 31 | loss | 2 |
| 50 | 3 | 7433257 | 8442946 | 135,849 | 241 | loss | 12 |
| 51 | 3 | 16634949 | 16749727 | 114,779 | 38 | loss | 2 |
| 52 | 3 | 53481315 | 53919417 | 438,103 | 87 | gain | 2 |
| 53 | 3 | 57052098 | 57163835 | 111,738 | 38 | loss | 2 |
| 54 | 3 | 62001931 | 62207780 | 205,850 | 63 | loss | 3 |
| 55 | 3 | 96164470 | 96324114 | 150,625 | 62 | loss | 5 |
| 56 | 3 | 102630095 | 102825632 | 195,538 | 57 | loss | 3 |
| 57 | 3 | 103417770 | 104326732 | 285,357 | 120 | loss | 8 |
| 58 | 3 | 109244550 | 109499513 | 254,964 | 49 | gain | 3 |
| 59 | 3 | 132263310 | 132426870 | 117,023 | 30 | loss | 11 |
| 60 | 3 | 134028482 | 134163268 | 134,787 | 42 | loss | 2 |
| 61 | 3 | 136934261 | 137073046 | 122,224 | 34 | loss | 4 |
| 62 | 3 | 137512720 | 137614332 | 101,613 | 24 | loss | 5 |
| 63 | 3 | 138535174 | 138641557 | 106,384 | 35 | loss | 2 |
| 64 | 3 | 161470038 | 161651595 | 181,558 | 44 | loss | 2 |
| 65 | 3 | 161755779 | 161899484 | 133,144 | 50 | loss | 3 |
| 66 | 3 | 162078846 | 162243480 | 104,221 | 41 | loss | 2 |
| 67 | 3 | 164004285 | 164295965 | 104,314 | 46 | gain | 5 |
| 68 | 3 | 204182428 | 204303130 | 118,182 | 29 | gain | 2 |
| 69 | 3 | 207430826 | 208018379 | 117,484 | 173 | loss | 4 |
| 70 | 3 | 213369816 | 213645115 | 103,243 | 67 | loss | 13 |
| 71 | 3 | 213819383 | 214133909 | 174,041 | 63 | loss | 8 |
| 72 | 3 | 216365248 | 216550479 | 185,232 | 46 | loss | 2 |
| 73 | 3 | 218568542 | 218717424 | 148,883 | 43 | loss | 2 |
| 74 | 3 | 218947523 | 219093917 | 135,368 | 50 | loss | 2 |
| 75 | 3 | 220246530 | 220407447 | 160,918 | 38 | loss | 2 |
| 76 | 3 | 220656591 | 221037310 | 120,022 | 78 | loss | 5 |
| 77 | 3 | 222525469 | 222748048 | 156,050 | 44 | loss | 10 |
| 78 | 3 | 223312694 | 224278204 | 147,054 | 104 | loss | 27 |
| 79 | 4 | 68779294 | 68942168 | 105,397 | 31 | loss | 10 |
| 80 | 4 | 76176907 | 76326642 | 149,736 | 45 | loss | 2 |
| 81 | 4 | 92817945 | 92977735 | 104,872 | 57 | loss | 11 |
| 82 | 4 | 111946218 | 112128525 | 124,582 | 83 | loss | 6 |
| 83 | 4 | 112936909 | 113154606 | 126,777 | 55 | loss | 15 |
| 84 | 4 | 116244031 | 116698658 | 119,855 | 124 | loss | 4 |
| 85 | 4 | 117568387 | 118011776 | 192,257 | 110 | loss | 8 |
| 86 | 4 | 118466452 | 118987294 | 162,835 | 138 | loss | 6 |
| 87 | 4 | 119063484 | 119166265 | 102,782 | 35 | loss | 2 |
| 88 | 5 | 5526844 | 5638078 | 107,700 | 52 | loss | 3 |
| 89 | 5 | 9603655 | 9828385 | 111,644 | 64 | loss | 6 |
| 90 | 5 | 12563777 | 12777646 | 213,870 | 33 | loss | 2 |
| 91 | 5 | 15973003 | 16230506 | 108,304 | 50 | loss | 10 |
| 92 | 5 | 16677746 | 16842873 | 151,702 | 60 | loss | 5 |
| 93 | 5 | 17200026 | 17342369 | 142,344 | 40 | loss | 2 |
| 94 | 5 | 17843227 | 18298637 | 139,412 | 92 | loss | 5 |
| 95 | 5 | 18928518 | 19064396 | 125,728 | 28 | loss | 2 |
| 96 | 5 | 36330170 | 36701778 | 142,743 | 84 | loss | 6 |
| 97 | 5 | 39181048 | 39289546 | 107,083 | 28 | loss | 5 |
| 98 | 5 | 40516067 | 41674534 | 161,409 | 156 | loss | 20 |
| 99 | 5 | 48233642 | 48424371 | 190,730 | 48 | loss | 2 |
| 100 | 5 | 58981975 | 59553761 | 562,217 | 156 | loss | 4 |
| 101 | 6 | 37325169 | 37451479 | 126,311 | 28 | gain | 2 |
| 102 | 6 | 51471221 | 51934454 | 421,617 | 92 | gain | 2 |
| 103 | 6 | 53995243 | 54169021 | 164,982 | 38 | gain | 2 |
| 104 | 6 | 64665847 | 64775497 | 109,651 | 20 | gain | 2 |
| 105 | 6 | 74004726 | 74151921 | 147,196 | 27 | gain | 3 |
| 106 | 6 | 75285216 | 75477224 | 105,372 | 34 | gain | 3 |
| 107 | 6 | 78155315 | 79396751 | 1,241,437 | 204 | gain | 2 |
| 108 | 6 | 113671647 | 113826884 | 155,238 | 45 | loss | 2 |
| 109 | 6 | 113900677 | 114031910 | 103,489 | 46 | loss | 4 |
| 110 | 6 | 114187523 | 114305162 | 110,890 | 34 | loss | 2 |
| 111 | 6 | 114711090 | 114890498 | 100,130 | 34 | loss | 22 |
| 112 | 6 | 114917900 | 115046716 | 128,817 | 35 | loss | 2 |
| 113 | 6 | 115324704 | 115766842 | 164,843 | 75 | loss | 12 |
| 114 | 6 | 115908289 | 117025755 | 139,343 | 154 | loss | 36 |
| 115 | 7 | 20340968 | 20691591 | 209,408 | 90 | loss | 2 |
| 116 | 7 | 21002815 | 21240846 | 105,741 | 64 | loss | 7 |
| 117 | 7 | 22342026 | 22705659 | 194,686 | 53 | gain | 5 |
| 118 | 7 | 32875841 | 33001901 | 110,257 | 31 | loss | 5 |
| 119 | 7 | 42327002 | 42431756 | 104,755 | 22 | loss | 2 |
| 120 | 7 | 83026370 | 83128721 | 102,352 | 31 | loss | 2 |
| 121 | 7 | 84623239 | 84738474 | 105,335 | 38 | loss | 3 |
| 122 | 7 | 98594746 | 98774110 | 179,365 | 42 | loss | 2 |
| 123 | 7 | 98896287 | 99551698 | 195,116 | 110 | loss | 10 |
| 124 | 8 | 2229149 | 2437064 | 173,186 | 31 | gain | 10 |
| 125 | 8 | 87802238 | 88083916 | 281,679 | 70 | loss | 2 |
| 126 | 8 | 88975019 | 90608988 | 124,008 | 194 | loss | 19 |
| 127 | 9 | 7231660 | 7456556 | 224,897 | 38 | gain | 2 |
| 128 | 9 | 9127137 | 9321384 | 172,959 | 33 | gain | 2 |
| 129 | 9 | 13265742 | 14325495 | 100,654 | 149 | loss | 30 |
| 130 | 9 | 14485051 | 14948677 | 225,058 | 139 | loss | 18 |
| 131 | 9 | 15253110 | 15654092 | 121,617 | 89 | loss | 9 |
| 132 | 9 | 77377746 | 77538846 | 141,271 | 18 | gain | 2 |
| 133 | 9 | 93880599 | 93995655 | 115,057 | 30 | loss | 2 |
| 134 | 10 | 5983222 | 6349615 | 366,394 | 67 | gain | 2 |
| 135 | 10 | 7722216 | 8101876 | 379,661 | 51 | gain | 2 |
| 136 | 10 | 32300354 | 33152569 | 852,216 | 197 | loss | 2 |
| 137 | 10 | 34408109 | 34847668 | 111,157 | 114 | loss | 4 |
| 138 | 10 | 42662880 | 43961987 | 127,998 | 139 | gain | 4 |
| 139 | 10 | 70548274 | 71182856 | 113,195 | 108 | gain-loss | 10 |
| 140 | 10 | 71207792 | 71383462 | 130,863 | 35 | gain | 2 |
| 141 | 10 | 84374095 | 84654824 | 108,285 | 88 | loss | 2 |
| 142 | 10 | 84761890 | 85022309 | 182,473 | 79 | loss | 6 |
| 143 | 10 | 85178675 | 85430393 | 251,719 | 90 | loss | 3 |
| 144 | 10 | 85689187 | 86439042 | 136,843 | 209 | loss | 15 |
| 145 | 11 | 21335420 | 21497980 | 147,762 | 42 | loss | 3 |
| 146 | 11 | 22360419 | 22472972 | 101,953 | 43 | loss | 2 |
| 147 | 11 | 22566832 | 22711206 | 144,375 | 33 | loss | 3 |
| 148 | 11 | 24106189 | 24252950 | 146,762 | 41 | loss | 2 |
| 149 | 11 | 24545528 | 24743504 | 120,919 | 63 | loss | 11 |
| 150 | 11 | 26566292 | 26852331 | 175,466 | 60 | loss | 6 |
| 151 | 11 | 27094063 | 27263221 | 135,605 | 21 | loss | 2 |
| 152 | 11 | 28114596 | 28221302 | 106,707 | 30 | loss | 2 |
| 153 | 11 | 33597626 | 33811826 | 139,208 | 53 | loss | 2 |
| 154 | 11 | 33880806 | 34377894 | 222,450 | 109 | loss | 14 |
| 155 | 11 | 38134537 | 38343040 | 188,593 | 58 | loss | 4 |
| 156 | 11 | 39516534 | 39691100 | 104,366 | 53 | loss | 5 |
| 157 | 11 | 43485422 | 43605006 | 114,160 | 33 | loss | 2 |
| 158 | 11 | 44031324 | 44361965 | 149,653 | 120 | loss | 5 |
| 159 | 11 | 49876846 | 50231458 | 104,978 | 69 | loss | 10 |
| 160 | 11 | 50460411 | 50604870 | 129,639 | 35 | loss | 2 |
| 161 | 11 | 50630769 | 50780342 | 119,492 | 48 | loss | 4 |
| 162 | 11 | 52273618 | 52422931 | 143,914 | 56 | loss | 2 |
| 163 | 11 | 54551089 | 54660249 | 104,460 | 52 | loss | 2 |
| 164 | 11 | 54722583 | 55069947 | 115,719 | 110 | loss | 11 |
| 165 | 11 | 55223012 | 55639035 | 132,876 | 105 | loss | 11 |
| 166 | 11 | 60583577 | 60698231 | 114,655 | 40 | loss | 2 |
| 167 | 11 | 61083935 | 61236378 | 152,444 | 46 | loss | 3 |
| 168 | 11 | 61761425 | 62022289 | 188,094 | 58 | loss | 5 |
| 169 | 12 | 3500281 | 3648368 | 116,265 | 50 | loss | 4 |
| 170 | 12 | 12297070 | 12407158 | 110,089 | 25 | gain | 2 |
| 171 | 12 | 35743933 | 35845891 | 101,959 | 23 | gain | 3 |
| 172 | 12 | 40012073 | 40198334 | 115,630 | 59 | loss | 4 |
| 173 | 12 | 40968298 | 41113570 | 106,872 | 42 | loss | 8 |
| 174 | 12 | 44888051 | 45146175 | 130,618 | 68 | loss | 8 |
| 175 | 12 | 47312033 | 47439316 | 106,448 | 50 | loss | 7 |
| 176 | 12 | 47457266 | 48078331 | 157,011 | 93 | loss | 11 |
| 177 | 12 | 48288842 | 48890159 | 104,570 | 137 | loss | 11 |
| 178 | 12 | 49104120 | 49701432 | 179,347 | 90 | loss | 15 |
| 179 | 12 | 77532875 | 77985369 | 213,043 | 124 | loss | 13 |
| 180 | 12 | 78216556 | 79070188 | 150,923 | 147 | loss | 33 |
| 181 | 13 | 41847162 | 42004340 | 157,179 | 47 | loss | 2 |
| 182 | 13 | 52996440 | 54394650 | 148,199 | 222 | loss | 32 |
| 183 | 13 | 54471235 | 54595840 | 109,181 | 43 | loss | 2 |
| 184 | 13 | 59438137 | 59545742 | 107,606 | 23 | loss | 3 |
| 185 | 13 | 66320924 | 66422625 | 101,702 | 30 | loss | 2 |
| 186 | 13 | 73129416 | 73242958 | 113,543 | 33 | loss | 2 |
| 187 | 13 | 74127955 | 74341617 | 136,514 | 57 | loss | 2 |
| 188 | 13 | 78015251 | 78272151 | 256,901 | 66 | loss | 2 |
| 189 | 14 | 559223 | 696973 | 137,751 | 44 | loss | 2 |
| 190 | 14 | 1186098 | 1320060 | 131,706 | 45 | loss | 2 |
| 191 | 14 | 7454425 | 7670760 | 134,956 | 57 | loss | 2 |
| 192 | 14 | 10563162 | 10975657 | 139,980 | 107 | loss | 7 |
| 193 | 14 | 12620310 | 13730156 | 128,464 | 101 | loss | 18 |
| 194 | 14 | 13995161 | 14265734 | 119,208 | 97 | loss | 4 |
| 195 | 14 | 34139846 | 34555098 | 115,924 | 123 | loss | 2 |
| 196 | 14 | 42923610 | 43031499 | 107,890 | 35 | loss | 3 |
| 197 | 14 | 48874683 | 49028743 | 100,645 | 44 | loss | 2 |
| 198 | 14 | 49973822 | 50430667 | 120,455 | 90 | loss | 8 |
| 199 | 14 | 51754928 | 51880604 | 125,677 | 33 | loss | 2 |
| 200 | 14 | 59107917 | 59412204 | 304,288 | 63 | loss | 2 |
| 201 | 14 | 62424849 | 62722187 | 237,574 | 74 | loss | 5 |
| 202 | 15 | 2479390 | 2956459 | 477,070 | 90 | gain | 2 |
| 203 | 15 | 3323204 | 3834521 | 511,318 | 98 | gain | 2 |
| 204 | 15 | 9310457 | 9706301 | 157,388 | 84 | gain | 2 |
| 205 | 15 | 10376429 | 11238787 | 319,159 | 155 | gain | 8 |
| 206 | 15 | 11542853 | 12070947 | 484,876 | 99 | gain | 2 |
| 207 | 15 | 45601527 | 45787694 | 181,238 | 80 | gain | 12 |
| 208 | 15 | 45814480 | 45929086 | 108,747 | 53 | loss | 4 |
| 209 | 15 | 50906930 | 51097039 | 190,110 | 59 | loss | 3 |
| 210 | 15 | 52789409 | 52903990 | 106,007 | 27 | loss | 2 |
| 211 | 15 | 74068333 | 74181404 | 113,072 | 32 | loss | 2 |
| 212 | 15 | 75365036 | 75494800 | 129,765 | 20 | loss | 2 |
| 213 | 15 | 77672081 | 77894780 | 147,458 | 62 | loss | 2 |
| 214 | 15 | 77925526 | 78155682 | 111,979 | 63 | loss | 2 |
| 215 | 15 | 80417733 | 80919408 | 280,780 | 89 | loss | 12 |
| 216 | 16 | 27364891 | 27592367 | 227,477 | 56 | gain | 2 |
| 217 | 16 | 43013057 | 43308744 | 295,688 | 55 | gain | 3 |
| 218 | 16 | 44992940 | 45731165 | 738,226 | 148 | gain | 2 |
| 219 | 16 | 46846135 | 48275215 | 100,829 | 266 | gain | 8 |
| 220 | 16 | 52755375 | 53299691 | 330,330 | 124 | gain | 2 |
| 221 | 16 | 54415646 | 54522787 | 107,142 | 19 | gain | 3 |
| 222 | 16 | 70469116 | 71715748 | 100,731 | 242 | loss | 28 |
| 223 | 17 | 21377148 | 21777153 | 184,303 | 74 | gain | 6 |
| 224 | 17 | 22618206 | 22727661 | 101,772 | 22 | gain | 2 |
| 225 | 17 | 25118986 | 25602718 | 424,975 | 92 | gain | 2 |
| 226 | 17 | 26254879 | 26433688 | 178,810 | 32 | gain | 2 |
| 227 | 17 | 26722671 | 27384709 | 230,360 | 137 | gain | 3 |
| 228 | 17 | 31030720 | 31460231 | 110,428 | 91 | gain | 3 |
| 229 | 17 | 35997119 | 36181354 | 116,301 | 35 | gain | 4 |
| 230 | 17 | 36538740 | 37453979 | 431,687 | 190 | gain | 2 |
| 231 | 17 | 38038454 | 38586177 | 544,309 | 99 | gain | 2 |
| 232 | 17 | 44197555 | 44314807 | 105,915 | 35 | loss | 3 |
| 233 | 17 | 44536710 | 44865308 | 205,631 | 74 | loss | 5 |
| 234 | 17 | 50809428 | 51095849 | 108,780 | 52 | loss | 7 |
| 235 | 17 | 60760254 | 60868849 | 105,532 | 43 | loss | 2 |
| 236 | 17 | 68357082 | 68460531 | 103,450 | 35 | loss | 2 |
| 237 | 17 | 69404138 | 69560911 | 156,774 | 54 | loss | 2 |
| 238 | 17 | 70443207 | 72279291 | 115,578 | 106 | loss | 33 |
| 239 | 18 | 12207155 | 12646915 | 439,761 | 105 | gain | 4 |
| 240 | 18 | 29349010 | 29565671 | 216,662 | 57 | loss | 2 |
| 241 | 18 | 46335629 | 46461475 | 125,847 | 29 | gain | 2 |
| 242 | 18 | 49572630 | 49718769 | 146,140 | 30 | gain | 2 |
| 243 | 18 | 50942580 | 51135973 | 114,414 | 27 | gain | 5 |
| 244 | 18 | 51570792 | 51885571 | 181,653 | 60 | gain | 4 |
| 245 | 18 | 65827669 | 66022203 | 142,918 | 44 | loss | 7 |
| 246 | 18 | 66313906 | 66473582 | 159,677 | 44 | loss | 2 |
| 247 | 18 | 66850236 | 67125667 | 275,432 | 88 | loss | 2 |
| 248 | 18 | 67248769 | 68601777 | 153,057 | 117 | loss | 24 |
| 249 | 19 | 48324183 | 48483364 | 107,898 | 60 | loss | 2 |
| 250 | 19 | 50378578 | 50520625 | 142,048 | 50 | loss | 2 |
| 251 | 19 | 52433188 | 52580066 | 143,849 | 43 | loss | 3 |
| 252 | 19 | 54131639 | 54454621 | 322,983 | 92 | loss | 2 |
| 253 | 19 | 55968471 | 56119901 | 151,431 | 37 | loss | 2 |
| 254 | 19 | 58099609 | 58264637 | 165,029 | 41 | loss | 2 |
| 255 | 19 | 58521305 | 59386934 | 181,634 | 163 | loss | 14 |
| 256 | 19 | 59601816 | 60446408 | 217,388 | 85 | loss | 21 |
| 257 | 20 | 1600100 | 1794903 | 194,804 | 36 | gain | 4 |
| 258 | 20 | 7447755 | 7729320 | 112,924 | 81 | loss | 4 |
| 259 | 20 | 7835350 | 8272487 | 300,624 | 89 | loss | 5 |
| 260 | 20 | 16642933 | 16980080 | 123,880 | 86 | loss | 8 |
| 261 | 20 | 33054516 | 33226497 | 171,982 | 43 | gain | 2 |
| 262 | 20 | 49288659 | 49455878 | 126,360 | 40 | loss | 4 |
| 263 | 20 | 49612638 | 51060804 | 141,675 | 261 | loss | 22 |
| 264 | 21 | 26610033 | 26853642 | 117,688 | 65 | gain | 3 |
| 265 | 21 | 39533129 | 39662187 | 100,310 | 53 | loss | 5 |
| 266 | 21 | 41466987 | 41623569 | 109,998 | 46 | loss | 3 |
| 267 | 21 | 41844846 | 41977304 | 129,271 | 28 | loss | 3 |
| 268 | 21 | 42278040 | 42584061 | 195,909 | 66 | loss | 3 |
| 269 | 21 | 43023084 | 43144070 | 111,669 | 28 | loss | 12 |
| 270 | 21 | 44551183 | 44693332 | 110,029 | 36 | loss | 6 |
| 271 | 21 | 44722788 | 45079414 | 137,423 | 95 | loss | 4 |
| 272 | 21 | 46183397 | 46441834 | 258,438 | 78 | loss | 2 |
| 273 | 21 | 46583788 | 46758987 | 128,816 | 53 | loss | 2 |
| 274 | 21 | 47711116 | 48121222 | 153,072 | 73 | loss | 10 |
| 275 | 21 | 48579849 | 48831283 | 113,933 | 38 | loss | 10 |
| 276 | 21 | 48863630 | 49995820 | 100,798 | 86 | loss | 32 |
| 277 | 22 | 42997377 | 43182266 | 106,903 | 50 | loss | 5 |
| 278 | 22 | 47990537 | 48442567 | 452,031 | 114 | loss | 3 |
| 279 | 22 | 48910953 | 49080000 | 108,609 | 59 | loss | 4 |
| 280 | 22 | 49795586 | 50828450 | 325,070 | 165 | loss | 26 |
| 281 | 23 | 21864 | 223488 | 201,625 | 42 | gain | 2 |
| 282 | 23 | 515426 | 688137 | 134,398 | 36 | loss | 4 |
| 283 | 23 | 27249024 | 27410257 | 112,493 | 36 | gain | 2 |
| 284 | 23 | 60939369 | 61557715 | 110,920 | 125 | loss | 13 |
| 285 | 24 | 19967 | 1380176 | 121,527 | 281 | loss | 24 |
| 286 | 24 | 1459984 | 1821735 | 145,610 | 65 | loss | 16 |
| 287 | 24 | 1951409 | 2116386 | 138,057 | 46 | loss | 3 |
| 288 | 24 | 2212686 | 2508661 | 209,702 | 73 | loss | 5 |
| 289 | 24 | 3432193 | 3858265 | 183,368 | 97 | loss | 5 |
| 290 | 24 | 9557054 | 9744913 | 140,548 | 53 | loss | 3 |
| 291 | 24 | 33396541 | 33568119 | 124,733 | 48 | loss | 2 |
| 292 | 24 | 35072102 | 35187700 | 106,805 | 30 | loss | 2 |
| 293 | 24 | 38215963 | 38342714 | 103,064 | 37 | loss | 2 |
| 294 | 24 | 40327423 | 42029819 | 105,446 | 129 | loss | 33 |
| 295 | 25 | 2270592 | 2410224 | 126,226 | 33 | loss | 2 |
| 296 | 25 | 2438979 | 2665072 | 104,247 | 45 | gain | 3 |
| 297 | 25 | 21294442 | 21839135 | 310,115 | 100 | gain | 3 |
| 298 | 25 | 30152043 | 30326533 | 163,806 | 32 | gain | 2 |
| 299 | 25 | 34527354 | 34937775 | 410,422 | 100 | loss | 2 |
| 300 | 25 | 44091721 | 44223414 | 131,694 | 44 | loss | 2 |
| 301 | 26 | 1054696 | 1288261 | 189,749 | 71 | loss | 4 |

Table S3 the detailed features of CNVRs on autosomes identified in Tibetan sheep

| **CNVR ID** | **Chr** | **Start(bp)** | **End(bp)** | **Length(bp)** | **No.SNP** | **Type** | **No.Samples** |
| --- | --- | --- | --- | --- | --- | --- | --- |
| 1 | 1 | 110377227 | 110530871 | 106,352 | 39 | gain | 6 |
| 2 | 1 | 132730487 | 132964053 | 125,513 | 50 | loss | 2 |
| 3 | 1 | 263107746 | 263257369 | 149,624 | 42 | loss | 2 |
| 4 | 2 | 748334 | 991124 | 156,010 | 72 | loss | 2 |
| 5 | 2 | 248779962 | 248896322 | 116,361 | 26 | loss | 4 |
| 6 | 3 | 30394 | 895462 | 112,036 | 119 | loss | 15 |
| 7 | 3 | 1013489 | 1295841 | 119,391 | 83 | loss | 3 |
| 8 | 3 | 1408687 | 1845573 | 103,505 | 70 | loss | 17 |
| 9 | 3 | 2353067 | 2718699 | 365,633 | 104 | loss | 2 |
| 10 | 3 | 2921398 | 3139238 | 122,096 | 77 | loss | 4 |
| 11 | 3 | 28207165 | 28322815 | 111,073 | 35 | loss | 2 |
| 12 | 3 | 220212548 | 220518683 | 188,144 | 59 | loss | 4 |
| 13 | 3 | 223384701 | 223681589 | 241,111 | 50 | loss | 4 |
| 14 | 4 | 92862327 | 92972731 | 110,405 | 46 | loss | 2 |
| 15 | 4 | 112423479 | 112616407 | 192,929 | 25 | gain | 2 |
| 16 | 5 | 5526844 | 5638078 | 111,235 | 52 | loss | 2 |
| 17 | 5 | 13050819 | 13152156 | 101,338 | 24 | loss | 2 |
| 18 | 5 | 18897055 | 19054245 | 157,191 | 32 | loss | 3 |
| 19 | 5 | 39181048 | 39289546 | 108,034 | 28 | loss | 4 |
| 20 | 6 | 116113666 | 116774122 | 163,319 | 67 | loss | 20 |
| 21 | 6 | 116842816 | 116982158 | 139,343 | 26 | loss | 2 |
| 22 | 7 | 22342026 | 22689889 | 268,218 | 50 | gain | 4 |
| 23 | 8 | 2222170 | 2418546 | 153,254 | 30 | gain | 17 |
| 24 | 8 | 32460894 | 33013854 | 552,064 | 124 | loss | 2 |
| 25 | 9 | 13308873 | 13758104 | 163,834 | 63 | loss | 4 |
| 26 | 9 | 13888048 | 14250061 | 207,707 | 105 | loss | 5 |
| 27 | 9 | 14511367 | 14636653 | 117,012 | 39 | loss | 2 |
| 28 | 10 | 70548274 | 71403573 | 247,420 | 158 | gain-loss | 22 |
| 29 | 10 | 71666243 | 71869192 | 122,597 | 49 | gain | 5 |
| 30 | 10 | 71921203 | 72247452 | 170,709 | 65 | gain | 6 |
| 31 | 10 | 85734857 | 85876677 | 103,000 | 49 | loss | 2 |
| 32 | 10 | 86044154 | 86439042 | 121,261 | 84 | loss | 2 |
| 33 | 11 | 33909668 | 34029369 | 119,702 | 37 | loss | 2 |
| 34 | 11 | 54936652 | 55063612 | 107,231 | 39 | loss | 5 |
| 35 | 12 | 41006699 | 41108437 | 101,739 | 34 | loss | 2 |
| 36 | 12 | 47566434 | 47789855 | 116,226 | 77 | loss | 3 |
| 37 | 12 | 48539835 | 48654056 | 104,570 | 28 | loss | 4 |
| 38 | 12 | 49264735 | 49576105 | 271,475 | 59 | loss | 11 |
| 39 | 12 | 77742766 | 77912057 | 169,292 | 47 | loss | 2 |
| 40 | 12 | 78384632 | 78613305 | 136,583 | 39 | loss | 19 |
| 41 | 13 | 53803953 | 54032485 | 136,338 | 64 | loss | 4 |
| 42 | 14 | 13157531 | 13501697 | 164,383 | 82 | loss | 4 |
| 43 | 15 | 12635257 | 12781253 | 139,207 | 21 | loss | 2 |
| 44 | 15 | 45601527 | 45787694 | 149,586 | 80 | gain | 13 |
| 45 | 16 | 49393522 | 49546850 | 153,329 | 30 | loss | 2 |
| 46 | 16 | 70546193 | 70689063 | 142,871 | 26 | loss | 2 |
| 47 | 16 | 71202218 | 71325376 | 123,159 | 21 | loss | 2 |
| 48 | 16 | 71551153 | 71715748 | 100,731 | 33 | loss | 8 |
| 49 | 17 | 70136327 | 70357696 | 221,370 | 34 | gain | 2 |
| 50 | 17 | 70595565 | 70753695 | 135,984 | 26 | loss | 7 |
| 51 | 17 | 71351032 | 71582959 | 231,928 | 47 | loss | 2 |
| 52 | 17 | 71640729 | 72279291 | 146,970 | 107 | loss | 4 |
| 53 | 18 | 67782220 | 67904766 | 122,547 | 20 | loss | 2 |
| 54 | 19 | 59601816 | 59830968 | 163,476 | 36 | loss | 3 |
| 55 | 20 | 27613000 | 27790143 | 116,889 | 42 | gain | 4 |
| 56 | 20 | 49999099 | 50607639 | 144,396 | 60 | gain-loss | 9 |
| 57 | 21 | 49468562 | 49814846 | 195,702 | 30 | loss | 14 |
| 58 | 22 | 1585531 | 1726160 | 140,630 | 25 | loss | 2 |
| 59 | 22 | 48046775 | 48152456 | 105,682 | 32 | loss | 2 |
| 60 | 22 | 49913684 | 50282156 | 186,437 | 46 | loss | 4 |
| 61 | 22 | 50348506 | 50569495 | 166,332 | 67 | loss | 4 |
| 62 | 23 | 21864 | 232643 | 191,337 | 43 | gain | 2 |
| 63 | 24 | 402305 | 624864 | 148,528 | 56 | loss | 12 |
| 64 | 24 | 791618 | 936845 | 135,713 | 39 | loss | 3 |
| 65 | 24 | 41178161 | 41604370 | 115,528 | 84 | loss | 7 |
| 66 | 24 | 41690640 | 42018485 | 327,846 | 41 | loss | 2 |

Table S4 the detailed features of CNVRs on autosomes after merger CNVs of three breeds

| **CNVR ID** | **Chr** | **Start(bp)** | **End(bp)** | **Length(bp)** | **No.SNP** | **Type** | **No.Samples** |
| --- | --- | --- | --- | --- | --- | --- | --- |
| 1 | 1 | 17218 | 1282340 | 147,366 | 268 | 1 | 67 |
| 2 | 1 | 1542440 | 2425229 | 196,024 | 235 | 1 | 23 |
| 3 | 1 | 2546815 | 3132186 | 109,305 | 169 | 1 | 14 |
| 4 | 1 | 3269952 | 3643248 | 143,689 | 113 | 1 | 6 |
| 5 | 1 | 5227297 | 5413967 | 131,333 | 66 | 1 | 2 |
| 6 | 1 | 7285572 | 7741112 | 169,225 | 125 | 1 | 2 |
| 7 | 1 | 15069418 | 15178777 | 109,360 | 32 | 1 | 2 |
| 8 | 1 | 17860628 | 18043156 | 182,529 | 67 | 1 | 2 |
| 9 | 1 | 20737305 | 20951770 | 111,398 | 58 | 1 | 8 |
| 10 | 1 | 27328554 | 28025524 | 125,170 | 174 | 1 | 14 |
| 11 | 1 | 28972525 | 29148388 | 108,967 | 55 | 1 | 2 |
| 12 | 1 | 48764405 | 49055973 | 195,416 | 48 | 3 | 4 |
| 13 | 1 | 79973027 | 80101789 | 120,486 | 21 | 3 | 2 |
| 14 | 1 | 103744512 | 103888057 | 137,843 | 34 | 1 | 4 |
| 15 | 1 | 104674851 | 104839975 | 107,876 | 47 | 1 | 2 |
| 16 | 1 | 105267629 | 105691805 | 140,443 | 77 | 1 | 12 |
| 17 | 1 | 109388555 | 109703706 | 112,664 | 80 | 1 | 2 |
| 18 | 1 | 110377227 | 110530871 | 106,352 | 39 | 3 | 9 |
| 19 | 1 | 110601315 | 110760127 | 158,813 | 41 | 1 | 2 |
| 20 | 1 | 121333493 | 121473502 | 105,126 | 67 | 1 | 3 |
| 21 | 1 | 132730487 | 132964053 | 125,513 | 50 | 1 | 2 |
| 22 | 1 | 148587073 | 148866436 | 119,955 | 60 | 3 | 9 |
| 23 | 1 | 158774791 | 158876978 | 102,188 | 20 | 3 | 2 |
| 24 | 1 | 189649273 | 189752265 | 100,113 | 36 | 1 | 7 |
| 25 | 1 | 201011832 | 201216289 | 204,458 | 55 | 1 | 7 |
| 26 | 1 | 252954232 | 253096396 | 142,165 | 40 | 1 | 3 |
| 27 | 1 | 253339822 | 253599999 | 217,537 | 77 | 1 | 2 |
| 28 | 1 | 260011540 | 260301261 | 130,013 | 92 | 1 | 7 |
| 29 | 1 | 260933730 | 263519919 | 151,974 | 530 | 1 | 45 |
| 30 | 1 | 263597994 | 264296166 | 132,424 | 197 | 1 | 15 |
| 31 | 1 | 266476893 | 266619856 | 142,964 | 54 | 1 | 2 |
| 32 | 1 | 270483710 | 270598507 | 114,798 | 35 | 1 | 2 |
| 33 | 1 | 271962142 | 272391202 | 139,523 | 107 | 1 | 7 |
| 34 | 1 | 275198084 | 275579536 | 230,623 | 106 | 1 | 15 |
| 35 | 2 | 17125 | 1993066 | 128,684 | 379 | 1 | 53 |
| 36 | 2 | 2874473 | 3218221 | 343,749 | 120 | 1 | 3 |
| 37 | 2 | 9202943 | 9712161 | 143,783 | 162 | 1 | 5 |
| 38 | 2 | 10266961 | 10376684 | 109,724 | 38 | 1 | 2 |
| 39 | 2 | 20015574 | 20164969 | 149,396 | 30 | 3 | 2 |
| 40 | 2 | 27486813 | 27645627 | 143,472 | 42 | 1 | 5 |
| 41 | 2 | 28087289 | 28409373 | 128,940 | 64 | 1 | 9 |
| 42 | 2 | 36682587 | 36982400 | 158,790 | 78 | 1 | 2 |
| 43 | 2 | 43132271 | 43450508 | 105,670 | 78 | 1 | 12 |
| 44 | 2 | 43557111 | 43736830 | 144,389 | 52 | 1 | 2 |
| 45 | 2 | 51437426 | 51552223 | 114,798 | 32 | 1 | 2 |
| 46 | 2 | 52495620 | 52641482 | 106,513 | 56 | 1 | 4 |
| 47 | 2 | 103719936 | 103988508 | 149,969 | 65 | 1 | 2 |
| 48 | 2 | 104166258 | 104455738 | 289,481 | 74 | 1 | 2 |
| 49 | 2 | 110010145 | 110310826 | 204,084 | 82 | 1 | 6 |
| 50 | 2 | 110612336 | 111254655 | 242,527 | 134 | 1 | 14 |
| 51 | 2 | 113263009 | 113386994 | 123,986 | 22 | 1 | 3 |
| 52 | 2 | 115896519 | 116130062 | 202,434 | 61 | 1 | 6 |
| 53 | 2 | 116674076 | 116859969 | 145,996 | 43 | 1 | 11 |
| 54 | 2 | 117097356 | 117342715 | 217,230 | 60 | 1 | 6 |
| 55 | 2 | 122385838 | 122869959 | 484,122 | 84 | 3 | 3 |
| 56 | 2 | 132818788 | 132940252 | 121,465 | 30 | 1 | 3 |
| 57 | 2 | 167762441 | 168046460 | 284,020 | 69 | 3 | 2 |
| 58 | 2 | 184594600 | 185268986 | 190,986 | 155 | 1 | 8 |
| 59 | 2 | 189571143 | 189739051 | 167,909 | 32 | 3 | 2 |
| 60 | 2 | 194252627 | 194563518 | 310,892 | 47 | 3 | 2 |
| 61 | 2 | 218798138 | 219376349 | 130,664 | 104 | 1 | 13 |
| 62 | 2 | 219781182 | 219990813 | 156,188 | 61 | 1 | 2 |
| 63 | 2 | 220088311 | 220539843 | 100,906 | 146 | 1 | 16 |
| 64 | 2 | 232234842 | 232486330 | 182,135 | 67 | 1 | 7 |
| 65 | 2 | 233076544 | 233631205 | 124,458 | 73 | 1 | 14 |
| 66 | 2 | 234304860 | 234447170 | 142,311 | 39 | 1 | 3 |
| 67 | 2 | 234745397 | 235142194 | 102,709 | 115 | 1 | 6 |
| 68 | 2 | 238515681 | 238871634 | 244,856 | 86 | 1 | 4 |
| 69 | 2 | 239119705 | 239243237 | 110,557 | 33 | 1 | 2 |
| 70 | 2 | 240735937 | 240992124 | 218,530 | 62 | 1 | 6 |
| 71 | 2 | 241478119 | 241700248 | 167,894 | 67 | 1 | 2 |
| 72 | 2 | 242723012 | 243204353 | 139,832 | 161 | 1 | 11 |
| 73 | 2 | 243353510 | 243603367 | 154,664 | 60 | 1 | 4 |
| 74 | 2 | 243697777 | 243885861 | 101,076 | 53 | 1 | 10 |
| 75 | 2 | 243983407 | 244097997 | 114,591 | 29 | 1 | 2 |
| 76 | 2 | 244800869 | 248981782 | 104,271 | 570 | 1 | 65 |
| 77 | 3 | 30394 | 4358207 | 113,295 | 973 | 1 | 99 |
| 78 | 3 | 4969959 | 5325867 | 278,435 | 92 | 1 | 7 |
| 79 | 3 | 5542371 | 5737015 | 139,003 | 77 | 1 | 5 |
| 80 | 3 | 6753855 | 6884511 | 130,657 | 31 | 1 | 7 |
| 81 | 3 | 7192808 | 8668862 | 142,899 | 241 | 1 | 27 |
| 82 | 3 | 8834343 | 8992087 | 150,502 | 43 | 1 | 3 |
| 83 | 3 | 9348876 | 9557043 | 208,168 | 59 | 1 | 2 |
| 84 | 3 | 11299226 | 11722361 | 195,055 | 110 | 1 | 2 |
| 85 | 3 | 13532931 | 13661207 | 108,525 | 38 | 1 | 2 |
| 86 | 3 | 13959873 | 14205181 | 101,984 | 54 | 1 | 5 |
| 87 | 3 | 16634949 | 16749727 | 114,779 | 38 | 1 | 2 |
| 88 | 3 | 17921712 | 18106593 | 129,161 | 50 | 1 | 5 |
| 89 | 3 | 18417040 | 18673107 | 241,647 | 81 | 1 | 2 |
| 90 | 3 | 19105792 | 19909972 | 804,181 | 197 | 1 | 2 |
| 91 | 3 | 28207165 | 28322815 | 111,073 | 35 | 1 | 2 |
| 92 | 3 | 53481315 | 53919417 | 438,103 | 87 | 3 | 2 |
| 93 | 3 | 57052098 | 57163835 | 111,738 | 38 | 1 | 2 |
| 94 | 3 | 57360189 | 57560618 | 126,063 | 44 | 1 | 4 |
| 95 | 3 | 62001931 | 62275376 | 205,850 | 93 | 1 | 5 |
| 96 | 3 | 80977828 | 81151449 | 173,622 | 42 | 1 | 2 |
| 97 | 3 | 88753699 | 88890614 | 136,916 | 27 | 3 | 3 |
| 98 | 3 | 96153092 | 96351441 | 104,201 | 73 | 1 | 15 |
| 99 | 3 | 97091525 | 97513405 | 112,700 | 121 | 1 | 4 |
| 100 | 3 | 102220378 | 102461636 | 241,259 | 65 | 1 | 2 |
| 101 | 3 | 102630095 | 102825632 | 195,538 | 57 | 1 | 11 |
| 102 | 3 | 103254925 | 104402177 | 121,706 | 305 | 1 | 21 |
| 103 | 3 | 109244550 | 109499513 | 245,467 | 49 | 3 | 6 |
| 104 | 3 | 120401806 | 120822876 | 421,071 | 89 | 3 | 2 |
| 105 | 3 | 132193852 | 132444146 | 106,917 | 55 | 1 | 18 |
| 106 | 3 | 133017090 | 133148483 | 131,394 | 40 | 1 | 2 |
| 107 | 3 | 133957365 | 134304682 | 134,787 | 98 | 1 | 8 |
| 108 | 3 | 136421489 | 136533303 | 102,196 | 34 | 1 | 3 |
| 109 | 3 | 136934261 | 137073046 | 122,224 | 34 | 1 | 7 |
| 110 | 3 | 137512720 | 137614332 | 101,613 | 24 | 1 | 10 |
| 111 | 3 | 138464202 | 138664939 | 114,865 | 67 | 1 | 7 |
| 112 | 3 | 158125037 | 158323478 | 125,966 | 37 | 3 | 4 |
| 113 | 3 | 161470038 | 161915835 | 142,685 | 113 | 1 | 12 |
| 114 | 3 | 162078846 | 162355920 | 104,221 | 71 | 1 | 5 |
| 115 | 3 | 163117882 | 163495532 | 196,088 | 89 | 1 | 2 |
| 116 | 3 | 164004285 | 164300378 | 110,824 | 46 | 2 | 13 |
| 117 | 3 | 178354420 | 178814735 | 407,028 | 132 | 1 | 4 |
| 118 | 3 | 179528439 | 179844384 | 315,946 | 88 | 1 | 2 |
| 119 | 3 | 180361995 | 180841024 | 206,609 | 72 | 1 | 5 |
| 120 | 3 | 204182428 | 204310421 | 118,182 | 31 | 3 | 4 |
| 121 | 3 | 207430826 | 208110083 | 109,960 | 185 | 1 | 8 |
| 122 | 3 | 210819726 | 211077975 | 185,488 | 62 | 1 | 5 |
| 123 | 3 | 212055171 | 212628102 | 111,970 | 152 | 1 | 4 |
| 124 | 3 | 212686337 | 212937950 | 162,122 | 59 | 1 | 6 |
| 125 | 3 | 213001360 | 214138526 | 174,041 | 182 | 1 | 46 |
| 126 | 3 | 214651731 | 214788769 | 137,039 | 39 | 1 | 3 |
| 127 | 3 | 214847067 | 214988410 | 141,344 | 46 | 1 | 2 |
| 128 | 3 | 216365248 | 216665636 | 185,232 | 70 | 1 | 3 |
| 129 | 3 | 216884648 | 216999700 | 101,913 | 34 | 1 | 3 |
| 130 | 3 | 217226491 | 218366042 | 107,317 | 330 | 1 | 7 |
| 131 | 3 | 218509635 | 221525942 | 139,345 | 294 | 1 | 38 |
| 132 | 3 | 222221815 | 222379116 | 157,302 | 52 | 1 | 2 |
| 133 | 3 | 222487959 | 222861323 | 226,875 | 59 | 1 | 22 |
| 134 | 3 | 223279489 | 224278204 | 131,845 | 197 | 1 | 65 |
| 135 | 4 | 32785607 | 32933344 | 147,738 | 30 | 3 | 2 |
| 136 | 4 | 65211323 | 65327602 | 116,280 | 32 | 1 | 2 |
| 137 | 4 | 68779294 | 68942168 | 105,397 | 33 | 1 | 36 |
| 138 | 4 | 76176907 | 76326642 | 149,736 | 45 | 1 | 2 |
| 139 | 4 | 76523880 | 76699046 | 139,726 | 53 | 1 | 5 |
| 140 | 4 | 77062020 | 77280714 | 116,445 | 76 | 1 | 4 |
| 141 | 4 | 92817945 | 92977735 | 110,405 | 60 | 1 | 22 |
| 142 | 4 | 98785415 | 98943045 | 157,631 | 52 | 1 | 2 |
| 143 | 4 | 111370333 | 112222888 | 203,947 | 251 | 1 | 16 |
| 144 | 4 | 112228559 | 112646391 | 192,929 | 80 | 3 | 6 |
| 145 | 4 | 112838743 | 113814706 | 102,559 | 241 | 1 | 36 |
| 146 | 4 | 116217849 | 119166265 | 102,782 | 491 | 1 | 37 |
| 147 | 5 | 1576810 | 1880404 | 212,614 | 85 | 1 | 2 |
| 148 | 5 | 2574241 | 2714621 | 104,830 | 46 | 1 | 2 |
| 149 | 5 | 3433954 | 5211755 | 107,393 | 411 | 1 | 10 |
| 150 | 5 | 5306065 | 5422203 | 116,139 | 43 | 1 | 2 |
| 151 | 5 | 5526844 | 5638078 | 111,235 | 52 | 1 | 7 |
| 152 | 5 | 8489379 | 8591881 | 102,503 | 18 | 1 | 2 |
| 153 | 5 | 9302707 | 10003364 | 146,747 | 222 | 1 | 12 |
| 154 | 5 | 10393615 | 10938908 | 175,878 | 141 | 1 | 4 |
| 155 | 5 | 12337169 | 13599186 | 113,702 | 308 | 1 | 12 |
| 156 | 5 | 14261191 | 14460794 | 199,604 | 58 | 1 | 2 |
| 157 | 5 | 15607771 | 15811834 | 204,064 | 51 | 1 | 2 |
| 158 | 5 | 15973003 | 16348376 | 108,304 | 84 | 1 | 26 |
| 159 | 5 | 16677746 | 19154728 | 125,728 | 584 | 1 | 47 |
| 160 | 5 | 19942912 | 20101166 | 110,196 | 41 | 1 | 6 |
| 161 | 5 | 35478809 | 35653159 | 117,893 | 69 | 1 | 3 |
| 162 | 5 | 35722404 | 35829833 | 107,430 | 27 | 1 | 2 |
| 163 | 5 | 36234400 | 36925460 | 124,056 | 154 | 1 | 11 |
| 164 | 5 | 38973944 | 39095642 | 109,002 | 17 | 3 | 2 |
| 165 | 5 | 39181048 | 39289546 | 108,034 | 28 | 1 | 9 |
| 166 | 5 | 40429310 | 41674534 | 161,409 | 236 | 1 | 44 |
| 167 | 5 | 48233642 | 48448480 | 175,748 | 48 | 1 | 5 |
| 168 | 5 | 58981975 | 59553761 | 562,217 | 156 | 1 | 5 |
| 169 | 5 | 59804966 | 59954532 | 100,114 | 31 | 1 | 3 |
| 170 | 5 | 82475727 | 83213540 | 115,768 | 108 | 3 | 6 |
| 171 | 6 | 9298021 | 9405460 | 107,440 | 23 | 3 | 2 |
| 172 | 6 | 31930104 | 32282237 | 127,537 | 74 | 3 | 4 |
| 173 | 6 | 33930366 | 34308545 | 123,747 | 78 | 3 | 3 |
| 174 | 6 | 37325169 | 37451479 | 126,311 | 28 | 3 | 7 |
| 175 | 6 | 51437554 | 51934454 | 421,617 | 99 | 3 | 7 |
| 176 | 6 | 53995243 | 54169021 | 164,982 | 38 | 3 | 2 |
| 177 | 6 | 64665847 | 64775497 | 109,651 | 20 | 3 | 4 |
| 178 | 6 | 74004726 | 74151921 | 134,028 | 27 | 3 | 6 |
| 179 | 6 | 75285216 | 76043555 | 758,340 | 146 | 2 | 6 |
| 180 | 6 | 78155315 | 80029344 | 305,648 | 317 | 2 | 13 |
| 181 | 6 | 80366205 | 80777028 | 228,403 | 91 | 3 | 2 |
| 182 | 6 | 85164829 | 85309224 | 131,708 | 38 | 3 | 4 |
| 183 | 6 | 102185688 | 102889568 | 703,881 | 232 | 1 | 3 |
| 184 | 6 | 112634642 | 112839180 | 204,539 | 55 | 1 | 3 |
| 185 | 6 | 113158368 | 113615021 | 138,395 | 82 | 1 | 8 |
| 186 | 6 | 113671647 | 114065869 | 103,489 | 79 | 1 | 16 |
| 187 | 6 | 114187523 | 114466560 | 102,293 | 55 | 1 | 4 |
| 188 | 6 | 114673197 | 115111078 | 129,973 | 86 | 1 | 49 |
| 189 | 6 | 115199394 | 115766842 | 164,843 | 82 | 1 | 26 |
| 190 | 6 | 115854155 | 117025755 | 139,343 | 154 | 1 | 92 |
| 191 | 7 | 13262576 | 13426522 | 108,904 | 40 | 1 | 4 |
| 192 | 7 | 20098704 | 20730415 | 209,408 | 161 | 1 | 5 |
| 193 | 7 | 21002815 | 21240846 | 105,741 | 69 | 1 | 12 |
| 194 | 7 | 22342026 | 22705659 | 107,899 | 53 | 3 | 17 |
| 195 | 7 | 32875841 | 33109128 | 100,968 | 51 | 1 | 19 |
| 196 | 7 | 33327726 | 33506754 | 124,799 | 61 | 1 | 4 |
| 197 | 7 | 42327002 | 42478104 | 151,103 | 35 | 1 | 3 |
| 198 | 7 | 82705699 | 82924327 | 154,139 | 68 | 1 | 2 |
| 199 | 7 | 83026370 | 83128721 | 102,352 | 31 | 1 | 3 |
| 200 | 7 | 84595003 | 84798923 | 105,335 | 62 | 1 | 7 |
| 201 | 7 | 84982372 | 85480686 | 159,917 | 128 | 1 | 5 |
| 202 | 7 | 96751386 | 97144392 | 377,148 | 107 | 1 | 2 |
| 203 | 7 | 97554990 | 98147160 | 592,171 | 148 | 1 | 3 |
| 204 | 7 | 98496118 | 99676548 | 155,774 | 305 | 1 | 23 |
| 205 | 8 | 2222170 | 2437064 | 153,254 | 32 | 3 | 45 |
| 206 | 8 | 32460894 | 33013854 | 552,064 | 124 | 1 | 2 |
| 207 | 8 | 34986855 | 35109159 | 122,305 | 33 | 3 | 2 |
| 208 | 8 | 83411549 | 83547691 | 136,143 | 43 | 1 | 2 |
| 209 | 8 | 87802238 | 88083916 | 281,679 | 70 | 1 | 2 |
| 210 | 8 | 88470881 | 90635782 | 124,008 | 413 | 1 | 47 |
| 211 | 9 | 7231660 | 7456556 | 224,897 | 38 | 3 | 2 |
| 212 | 9 | 9127137 | 9321384 | 172,959 | 33 | 3 | 2 |
| 213 | 9 | 10971394 | 11162085 | 190,692 | 34 | 3 | 2 |
| 214 | 9 | 13193845 | 16596810 | 133,174 | 900 | 1 | 83 |
| 215 | 9 | 20134758 | 20262957 | 128,200 | 36 | 1 | 2 |
| 216 | 9 | 63086070 | 63406906 | 320,837 | 69 | 3 | 2 |
| 217 | 9 | 77377746 | 77563420 | 141,271 | 20 | 3 | 4 |
| 218 | 9 | 93844970 | 94539303 | 115,057 | 195 | 1 | 10 |
| 219 | 10 | 5983222 | 6548405 | 366,394 | 100 | 3 | 3 |
| 220 | 10 | 6731527 | 7121419 | 112,588 | 63 | 3 | 2 |
| 221 | 10 | 7362107 | 8173096 | 379,661 | 122 | 3 | 8 |
| 222 | 10 | 21498197 | 21720606 | 222,410 | 78 | 1 | 2 |
| 223 | 10 | 32300354 | 33283953 | 113,781 | 233 | 1 | 5 |
| 224 | 10 | 34408109 | 34870291 | 111,157 | 124 | 1 | 8 |
| 225 | 10 | 35390498 | 35728094 | 238,626 | 70 | 1 | 2 |
| 226 | 10 | 35838530 | 36338933 | 500,404 | 109 | 1 | 4 |
| 227 | 10 | 36847123 | 36959483 | 112,361 | 34 | 1 | 2 |
| 228 | 10 | 38266408 | 38522127 | 136,706 | 41 | 3 | 3 |
| 229 | 10 | 38630143 | 39010516 | 380,374 | 54 | 3 | 2 |
| 230 | 10 | 39448177 | 39554305 | 106,129 | 22 | 3 | 2 |
| 231 | 10 | 42662880 | 43961987 | 127,998 | 139 | 3 | 9 |
| 232 | 10 | 44959202 | 45404298 | 445,097 | 81 | 3 | 2 |
| 233 | 10 | 70548274 | 71404451 | 116,163 | 158 | 2 | 54 |
| 234 | 10 | 71666243 | 71869192 | 173,737 | 49 | 3 | 8 |
| 235 | 10 | 71921203 | 72247452 | 170,709 | 65 | 3 | 12 |
| 236 | 10 | 84374095 | 85022309 | 182,473 | 88 | 1 | 30 |
| 237 | 10 | 85178675 | 86439042 | 113,166 | 209 | 1 | 46 |
| 238 | 11 | 11167913 | 11287843 | 119,931 | 37 | 1 | 2 |
| 239 | 11 | 18080459 | 18238141 | 144,707 | 49 | 1 | 3 |
| 240 | 11 | 19474851 | 20175974 | 101,775 | 205 | 1 | 4 |
| 241 | 11 | 21216407 | 21716773 | 147,762 | 142 | 1 | 9 |
| 242 | 11 | 21868581 | 22113776 | 141,765 | 73 | 1 | 4 |
| 243 | 11 | 22218565 | 22472972 | 101,953 | 83 | 1 | 11 |
| 244 | 11 | 22566832 | 22728568 | 144,375 | 35 | 1 | 6 |
| 245 | 11 | 23195819 | 23505479 | 140,731 | 87 | 1 | 4 |
| 246 | 11 | 24106189 | 24252950 | 133,684 | 41 | 1 | 5 |
| 247 | 11 | 24545528 | 24743504 | 120,919 | 63 | 1 | 19 |
| 248 | 11 | 26550012 | 27498215 | 134,106 | 142 | 1 | 24 |
| 249 | 11 | 27816888 | 28062254 | 133,633 | 68 | 1 | 5 |
| 250 | 11 | 28114596 | 28221302 | 106,707 | 30 | 1 | 2 |
| 251 | 11 | 32607896 | 33066539 | 181,334 | 101 | 1 | 2 |
| 252 | 11 | 33597626 | 34597636 | 138,143 | 109 | 1 | 45 |
| 253 | 11 | 34738597 | 34894423 | 104,938 | 49 | 1 | 5 |
| 254 | 11 | 37398200 | 37501773 | 103,574 | 22 | 1 | 2 |
| 255 | 11 | 38134537 | 38343040 | 188,593 | 58 | 1 | 4 |
| 256 | 11 | 38554499 | 38787419 | 138,031 | 73 | 1 | 2 |
| 257 | 11 | 39516534 | 39691100 | 104,366 | 53 | 1 | 6 |
| 258 | 11 | 41648968 | 41796063 | 115,195 | 43 | 1 | 7 |
| 259 | 11 | 42280234 | 42405945 | 125,712 | 45 | 1 | 2 |
| 260 | 11 | 43485422 | 43605006 | 114,160 | 33 | 1 | 2 |
| 261 | 11 | 44031324 | 44361965 | 149,653 | 120 | 1 | 6 |
| 262 | 11 | 47175399 | 47305094 | 110,617 | 41 | 1 | 3 |
| 263 | 11 | 48924471 | 49038684 | 110,530 | 37 | 1 | 2 |
| 264 | 11 | 49498051 | 49688184 | 190,134 | 55 | 1 | 2 |
| 265 | 11 | 49764393 | 50838326 | 119,492 | 265 | 1 | 37 |
| 266 | 11 | 51406283 | 51570512 | 147,370 | 60 | 1 | 4 |
| 267 | 11 | 52226107 | 52550458 | 143,914 | 120 | 1 | 8 |
| 268 | 11 | 52906832 | 53074284 | 167,453 | 58 | 1 | 2 |
| 269 | 11 | 54258558 | 54467069 | 195,980 | 64 | 1 | 6 |
| 270 | 11 | 54529615 | 55069947 | 107,231 | 192 | 1 | 38 |
| 271 | 11 | 55223012 | 55673721 | 117,314 | 105 | 1 | 27 |
| 272 | 11 | 55905371 | 56372885 | 155,665 | 145 | 1 | 4 |
| 273 | 11 | 56630543 | 57068226 | 170,254 | 69 | 1 | 7 |
| 274 | 11 | 60581685 | 60774789 | 131,261 | 60 | 1 | 7 |
| 275 | 11 | 60981089 | 61302775 | 122,196 | 75 | 1 | 15 |
| 276 | 11 | 61534975 | 62043545 | 170,268 | 76 | 1 | 21 |
| 277 | 12 | 1190747 | 1315546 | 124,800 | 48 | 1 | 2 |
| 278 | 12 | 2030972 | 2156494 | 119,174 | 44 | 1 | 2 |
| 279 | 12 | 2499928 | 2767564 | 100,753 | 70 | 1 | 2 |
| 280 | 12 | 3500281 | 3648368 | 113,957 | 50 | 1 | 14 |
| 281 | 12 | 12297070 | 12451644 | 110,089 | 37 | 3 | 3 |
| 282 | 12 | 25010450 | 25204961 | 171,298 | 78 | 1 | 2 |
| 283 | 12 | 35743933 | 35845891 | 101,959 | 23 | 3 | 8 |
| 284 | 12 | 40012073 | 40198334 | 115,630 | 59 | 1 | 9 |
| 285 | 12 | 40946885 | 41116411 | 101,739 | 44 | 1 | 20 |
| 286 | 12 | 41763886 | 41972201 | 186,185 | 53 | 1 | 5 |
| 287 | 12 | 42211434 | 42486394 | 144,654 | 68 | 1 | 9 |
| 288 | 12 | 43774586 | 44052246 | 249,499 | 75 | 1 | 3 |
| 289 | 12 | 44888051 | 45592652 | 270,321 | 200 | 1 | 28 |
| 290 | 12 | 47164666 | 48917048 | 118,687 | 458 | 1 | 62 |
| 291 | 12 | 49002167 | 49901432 | 179,347 | 118 | 1 | 52 |
| 292 | 12 | 61964043 | 62127531 | 163,489 | 40 | 3 | 2 |
| 293 | 12 | 75166383 | 75290670 | 124,288 | 30 | 1 | 3 |
| 294 | 12 | 77484498 | 77985369 | 169,292 | 129 | 1 | 32 |
| 295 | 12 | 78216556 | 79070188 | 150,923 | 147 | 1 | 81 |
| 296 | 13 | 41847162 | 42004340 | 150,384 | 47 | 1 | 3 |
| 297 | 13 | 45193041 | 45758178 | 101,406 | 187 | 1 | 2 |
| 298 | 13 | 52996440 | 54985635 | 109,181 | 520 | 1 | 76 |
| 299 | 13 | 59438137 | 59764923 | 126,376 | 64 | 1 | 7 |
| 300 | 13 | 60370980 | 60533120 | 135,443 | 37 | 1 | 4 |
| 301 | 13 | 66320924 | 66422625 | 101,702 | 30 | 1 | 2 |
| 302 | 13 | 71723830 | 71834402 | 110,573 | 36 | 1 | 2 |
| 303 | 13 | 73129416 | 73242958 | 113,543 | 33 | 1 | 2 |
| 304 | 13 | 74127955 | 74341617 | 118,588 | 57 | 1 | 10 |
| 305 | 13 | 78015251 | 78272151 | 256,901 | 66 | 1 | 2 |
| 306 | 13 | 78374859 | 78475773 | 100,915 | 30 | 1 | 2 |
| 307 | 13 | 78742887 | 78852781 | 104,612 | 30 | 1 | 4 |
| 308 | 13 | 78882747 | 79047581 | 164,835 | 40 | 1 | 2 |
| 309 | 13 | 81267177 | 81406516 | 102,063 | 41 | 1 | 2 |
| 310 | 13 | 82653401 | 83022168 | 368,768 | 90 | 1 | 2 |
| 311 | 14 | 483895 | 1320060 | 116,067 | 242 | 1 | 11 |
| 312 | 14 | 1811644 | 2015710 | 117,087 | 55 | 1 | 11 |
| 313 | 14 | 7416410 | 7874805 | 134,956 | 118 | 1 | 7 |
| 314 | 14 | 10433812 | 14324839 | 151,939 | 512 | 1 | 56 |
| 315 | 14 | 18456385 | 18607834 | 122,189 | 44 | 1 | 2 |
| 316 | 14 | 25299417 | 25413664 | 114,248 | 37 | 1 | 2 |
| 317 | 14 | 33394829 | 33631168 | 106,643 | 66 | 1 | 2 |
| 318 | 14 | 33892398 | 34805062 | 108,505 | 123 | 1 | 25 |
| 319 | 14 | 42888243 | 43590748 | 107,890 | 179 | 1 | 7 |
| 320 | 14 | 44840080 | 44947670 | 107,591 | 36 | 1 | 2 |
| 321 | 14 | 45459317 | 45893975 | 306,739 | 161 | 1 | 4 |
| 322 | 14 | 47165085 | 47672055 | 506,971 | 123 | 1 | 2 |
| 323 | 14 | 48002289 | 48388672 | 354,289 | 105 | 1 | 2 |
| 324 | 14 | 48738235 | 49223740 | 165,309 | 137 | 1 | 11 |
| 325 | 14 | 49597174 | 49778524 | 181,351 | 51 | 1 | 2 |
| 326 | 14 | 49934777 | 50507077 | 120,455 | 134 | 1 | 20 |
| 327 | 14 | 50888672 | 51076943 | 149,960 | 63 | 1 | 2 |
| 328 | 14 | 51575247 | 52051702 | 125,677 | 84 | 1 | 6 |
| 329 | 14 | 52369849 | 52957964 | 148,357 | 152 | 1 | 9 |
| 330 | 14 | 54891002 | 55242725 | 109,921 | 99 | 1 | 5 |
| 331 | 14 | 55800418 | 55961366 | 148,638 | 56 | 1 | 2 |
| 332 | 14 | 59107917 | 59610579 | 133,806 | 133 | 1 | 17 |
| 333 | 14 | 62424849 | 62722187 | 148,423 | 74 | 1 | 18 |
| 334 | 15 | 2260784 | 2956459 | 101,108 | 120 | 3 | 9 |
| 335 | 15 | 3323204 | 3834521 | 511,318 | 98 | 3 | 2 |
| 336 | 15 | 9310457 | 9706301 | 157,388 | 84 | 3 | 3 |
| 337 | 15 | 10274423 | 11238787 | 319,159 | 155 | 3 | 23 |
| 338 | 15 | 11542853 | 12101107 | 201,993 | 99 | 3 | 6 |
| 339 | 15 | 12635257 | 12781253 | 139,207 | 21 | 1 | 2 |
| 340 | 15 | 27361124 | 27563048 | 195,168 | 72 | 1 | 2 |
| 341 | 15 | 43725680 | 43898728 | 163,578 | 51 | 1 | 3 |
| 342 | 15 | 45601527 | 45787694 | 149,586 | 80 | 3 | 47 |
| 343 | 15 | 45814480 | 46061865 | 108,747 | 93 | 1 | 9 |
| 344 | 15 | 50342943 | 50486417 | 143,475 | 44 | 1 | 2 |
| 345 | 15 | 50906930 | 51097039 | 190,110 | 59 | 1 | 4 |
| 346 | 15 | 52789409 | 52958533 | 169,125 | 50 | 1 | 6 |
| 347 | 15 | 53733768 | 53839596 | 105,829 | 24 | 1 | 2 |
| 348 | 15 | 54203565 | 54349173 | 145,609 | 54 | 1 | 2 |
| 349 | 15 | 54576023 | 54755666 | 144,076 | 60 | 1 | 6 |
| 350 | 15 | 67736788 | 67865703 | 128,916 | 28 | 3 | 2 |
| 351 | 15 | 72800500 | 72926314 | 125,815 | 36 | 1 | 2 |
| 352 | 15 | 74068333 | 74199604 | 113,072 | 38 | 1 | 6 |
| 353 | 15 | 74475270 | 74645248 | 103,810 | 49 | 1 | 4 |
| 354 | 15 | 75297169 | 75625948 | 129,765 | 72 | 1 | 5 |
| 355 | 15 | 77672081 | 78220052 | 111,979 | 155 | 1 | 9 |
| 356 | 15 | 79028361 | 79154352 | 121,876 | 46 | 1 | 2 |
| 357 | 15 | 80410579 | 80919408 | 165,014 | 89 | 1 | 34 |
| 358 | 16 | 473598 | 797065 | 323,468 | 96 | 1 | 2 |
| 359 | 16 | 27364891 | 27690818 | 318,741 | 74 | 3 | 4 |
| 360 | 16 | 43013057 | 43308744 | 295,688 | 55 | 3 | 3 |
| 361 | 16 | 44992940 | 45794856 | 143,354 | 148 | 3 | 5 |
| 362 | 16 | 46846135 | 48389206 | 100,829 | 266 | 3 | 19 |
| 363 | 16 | 49393522 | 49546850 | 153,329 | 30 | 1 | 2 |
| 364 | 16 | 52755375 | 53299691 | 330,330 | 124 | 3 | 4 |
| 365 | 16 | 54415646 | 54522787 | 107,142 | 19 | 3 | 3 |
| 366 | 16 | 69203274 | 69421991 | 115,815 | 62 | 1 | 2 |
| 367 | 16 | 69943027 | 70206922 | 121,619 | 92 | 1 | 7 |
| 368 | 16 | 70469116 | 71715748 | 100,731 | 242 | 1 | 74 |
| 369 | 17 | 21377148 | 21937011 | 122,644 | 96 | 3 | 13 |
| 370 | 17 | 22618206 | 22727661 | 101,772 | 22 | 3 | 3 |
| 371 | 17 | 23381788 | 23496203 | 114,416 | 28 | 3 | 2 |
| 372 | 17 | 25118986 | 25602718 | 424,975 | 92 | 3 | 3 |
| 373 | 17 | 26254879 | 26433688 | 178,810 | 32 | 3 | 2 |
| 374 | 17 | 26713614 | 27384709 | 101,391 | 137 | 3 | 14 |
| 375 | 17 | 31030720 | 31460231 | 110,428 | 91 | 3 | 4 |
| 376 | 17 | 34277999 | 34385859 | 107,861 | 24 | 3 | 2 |
| 377 | 17 | 35997119 | 36181354 | 116,301 | 35 | 3 | 4 |
| 378 | 17 | 36538740 | 37453979 | 431,687 | 190 | 3 | 3 |
| 379 | 17 | 38038454 | 38586177 | 544,309 | 99 | 3 | 2 |
| 380 | 17 | 44024856 | 44894126 | 205,631 | 249 | 1 | 21 |
| 381 | 17 | 50809428 | 51095849 | 101,182 | 52 | 1 | 19 |
| 382 | 17 | 52107896 | 52286741 | 178,846 | 34 | 1 | 4 |
| 383 | 17 | 60237606 | 60404477 | 155,837 | 69 | 1 | 2 |
| 384 | 17 | 60760254 | 60868849 | 105,532 | 43 | 1 | 2 |
| 385 | 17 | 62096410 | 62247317 | 141,946 | 43 | 1 | 8 |
| 386 | 17 | 63031657 | 63257791 | 226,135 | 52 | 1 | 2 |
| 387 | 17 | 63471418 | 63662570 | 111,348 | 64 | 1 | 2 |
| 388 | 17 | 68357082 | 68470428 | 103,450 | 37 | 1 | 4 |
| 389 | 17 | 68806525 | 69560911 | 156,774 | 227 | 1 | 6 |
| 390 | 17 | 69710844 | 69882461 | 126,085 | 71 | 1 | 4 |
| 391 | 17 | 70136327 | 70357696 | 221,370 | 34 | 3 | 3 |
| 392 | 17 | 70443207 | 72279291 | 115,578 | 391 | 1 | 81 |
| 393 | 18 | 1802043 | 2023593 | 113,664 | 64 | 1 | 4 |
| 394 | 18 | 12207155 | 12653998 | 439,761 | 106 | 3 | 7 |
| 395 | 18 | 18848018 | 18954126 | 106,109 | 19 | 3 | 2 |
| 396 | 18 | 19488042 | 19599474 | 111,433 | 39 | 1 | 3 |
| 397 | 18 | 19904707 | 20085240 | 144,443 | 46 | 1 | 5 |
| 398 | 18 | 29349010 | 29565671 | 216,662 | 57 | 1 | 4 |
| 399 | 18 | 31786788 | 32136593 | 320,778 | 118 | 1 | 5 |
| 400 | 18 | 32365541 | 32487822 | 122,282 | 25 | 1 | 6 |
| 401 | 18 | 32666161 | 32846550 | 120,600 | 42 | 1 | 4 |
| 402 | 18 | 46335629 | 46461475 | 125,847 | 29 | 3 | 2 |
| 403 | 18 | 49572630 | 49718769 | 146,140 | 30 | 3 | 2 |
| 404 | 18 | 50942580 | 51135973 | 114,414 | 27 | 3 | 8 |
| 405 | 18 | 51570792 | 51885571 | 181,653 | 60 | 3 | 5 |
| 406 | 18 | 63147533 | 63386051 | 218,151 | 60 | 1 | 4 |
| 407 | 18 | 63830156 | 63946456 | 109,059 | 35 | 1 | 5 |
| 408 | 18 | 64623786 | 66645322 | 137,555 | 446 | 1 | 21 |
| 409 | 18 | 66850236 | 68601777 | 100,271 | 134 | 1 | 54 |
| 410 | 19 | 11368559 | 11524505 | 155,947 | 49 | 1 | 2 |
| 411 | 19 | 47122415 | 47255723 | 133,309 | 32 | 1 | 2 |
| 412 | 19 | 48324183 | 48976603 | 102,910 | 194 | 1 | 10 |
| 413 | 19 | 49651397 | 50168046 | 241,729 | 112 | 1 | 15 |
| 414 | 19 | 50282691 | 50612222 | 142,048 | 104 | 1 | 11 |
| 415 | 19 | 50792800 | 50965294 | 149,415 | 55 | 1 | 5 |
| 416 | 19 | 51141275 | 51403316 | 103,720 | 104 | 1 | 8 |
| 417 | 19 | 52428671 | 52580066 | 143,849 | 45 | 1 | 20 |
| 418 | 19 | 54131639 | 54454621 | 130,318 | 92 | 1 | 10 |
| 419 | 19 | 55968471 | 56126158 | 132,629 | 37 | 1 | 8 |
| 420 | 19 | 57232845 | 57395959 | 109,219 | 41 | 1 | 3 |
| 421 | 19 | 57748802 | 57927983 | 179,182 | 54 | 1 | 2 |
| 422 | 19 | 58099609 | 58288122 | 165,029 | 47 | 1 | 3 |
| 423 | 19 | 58521305 | 60446408 | 217,388 | 416 | 1 | 59 |
| 424 | 20 | 1600100 | 1794903 | 194,804 | 36 | 3 | 4 |
| 425 | 20 | 7447755 | 7729320 | 112,924 | 81 | 1 | 8 |
| 426 | 20 | 7835350 | 8446449 | 102,394 | 89 | 1 | 16 |
| 427 | 20 | 8593363 | 8722533 | 118,363 | 28 | 1 | 2 |
| 428 | 20 | 9396217 | 9599768 | 111,960 | 47 | 1 | 4 |
| 429 | 20 | 10937086 | 11080463 | 110,331 | 42 | 1 | 3 |
| 430 | 20 | 15415171 | 15566192 | 140,896 | 37 | 1 | 2 |
| 431 | 20 | 16642933 | 16980080 | 123,880 | 86 | 1 | 18 |
| 432 | 20 | 17420003 | 17756193 | 336,191 | 72 | 1 | 2 |
| 433 | 20 | 27613000 | 27790143 | 116,889 | 42 | 3 | 10 |
| 434 | 20 | 28390615 | 28541787 | 117,407 | 30 | 3 | 5 |
| 435 | 20 | 33054516 | 33226497 | 171,982 | 43 | 3 | 3 |
| 436 | 20 | 49288659 | 49455878 | 126,360 | 40 | 1 | 6 |
| 437 | 20 | 49612638 | 51159416 | 141,675 | 261 | 2 | 52 |
| 438 | 21 | 26610033 | 26853642 | 117,688 | 65 | 3 | 3 |
| 439 | 21 | 35682186 | 35796243 | 114,058 | 36 | 1 | 2 |
| 440 | 21 | 39533129 | 39662187 | 102,898 | 53 | 1 | 14 |
| 441 | 21 | 40312864 | 40429486 | 107,096 | 42 | 1 | 2 |
| 442 | 21 | 41424501 | 41989188 | 129,271 | 142 | 1 | 12 |
| 443 | 21 | 42110775 | 42735679 | 102,429 | 156 | 1 | 11 |
| 444 | 21 | 43023084 | 43403450 | 111,669 | 102 | 1 | 23 |
| 445 | 21 | 44149257 | 44350445 | 201,189 | 48 | 1 | 3 |
| 446 | 21 | 44468253 | 50055911 | 100,798 | 538 | 1 | 90 |
| 447 | 22 | 1383367 | 2246347 | 106,822 | 140 | 2 | 8 |
| 448 | 22 | 20954454 | 21243674 | 284,501 | 79 | 1 | 4 |
| 449 | 22 | 23103977 | 23429477 | 111,074 | 92 | 1 | 6 |
| 450 | 22 | 42958926 | 43189643 | 130,915 | 57 | 1 | 15 |
| 451 | 22 | 47990537 | 48442567 | 105,682 | 114 | 1 | 10 |
| 452 | 22 | 48910953 | 49122945 | 107,392 | 59 | 1 | 8 |
| 453 | 22 | 49384312 | 50828450 | 222,762 | 282 | 1 | 71 |
| 454 | 23 | 21864 | 232643 | 191,337 | 43 | 3 | 4 |
| 455 | 23 | 515426 | 848215 | 107,748 | 83 | 1 | 13 |
| 456 | 23 | 988122 | 1228581 | 102,097 | 64 | 1 | 4 |
| 457 | 23 | 2185050 | 2351416 | 149,337 | 51 | 1 | 2 |
| 458 | 23 | 27249024 | 27410257 | 112,493 | 36 | 3 | 2 |
| 459 | 23 | 46291143 | 46424743 | 127,749 | 47 | 1 | 2 |
| 460 | 23 | 48672482 | 48798242 | 125,761 | 32 | 1 | 2 |
| 461 | 23 | 60207975 | 60752989 | 545,015 | 170 | 1 | 2 |
| 462 | 23 | 60939369 | 61873507 | 110,920 | 219 | 1 | 28 |
| 463 | 24 | 19967 | 1380176 | 121,527 | 382 | 1 | 73 |
| 464 | 24 | 1454321 | 1821735 | 145,610 | 82 | 1 | 42 |
| 465 | 24 | 1927823 | 2508661 | 209,702 | 136 | 1 | 28 |
| 466 | 24 | 2938579 | 3071910 | 133,332 | 68 | 1 | 2 |
| 467 | 24 | 3432193 | 4128679 | 107,159 | 104 | 1 | 19 |
| 468 | 24 | 7634259 | 7780633 | 100,417 | 38 | 1 | 5 |
| 469 | 24 | 9557054 | 9744913 | 140,548 | 53 | 1 | 3 |
| 470 | 24 | 24784697 | 25055998 | 271,302 | 76 | 1 | 3 |
| 471 | 24 | 26369853 | 26565757 | 195,905 | 66 | 1 | 2 |
| 472 | 24 | 26875194 | 27237559 | 362,366 | 101 | 1 | 2 |
| 473 | 24 | 33396541 | 33751167 | 112,559 | 103 | 1 | 6 |
| 474 | 24 | 34368526 | 34948696 | 182,696 | 56 | 1 | 5 |
| 475 | 24 | 35017019 | 35187700 | 106,805 | 40 | 1 | 5 |
| 476 | 24 | 35748028 | 35872661 | 124,634 | 25 | 1 | 3 |
| 477 | 24 | 37177842 | 38361294 | 109,781 | 331 | 1 | 25 |
| 478 | 24 | 38984006 | 39170476 | 109,818 | 60 | 1 | 8 |
| 479 | 24 | 40327423 | 42029819 | 105,446 | 191 | 1 | 79 |
| 480 | 25 | 2258259 | 2665072 | 104,247 | 45 | 2 | 8 |
| 481 | 25 | 13262379 | 13414827 | 152,449 | 47 | 1 | 2 |
| 482 | 25 | 20407519 | 20673498 | 265,980 | 54 | 3 | 2 |
| 483 | 25 | 21294442 | 21839135 | 310,115 | 100 | 3 | 3 |
| 484 | 25 | 27187968 | 27842576 | 116,499 | 212 | 1 | 6 |
| 485 | 25 | 30152043 | 30326533 | 163,806 | 32 | 3 | 3 |
| 486 | 25 | 34527354 | 34947355 | 118,241 | 100 | 1 | 7 |
| 487 | 25 | 44091721 | 44223414 | 131,694 | 44 | 1 | 2 |
| 488 | 26 | 425861 | 975890 | 117,833 | 140 | 1 | 5 |
| 489 | 26 | 1054696 | 1299859 | 189,749 | 75 | 1 | 7 |
| 490 | 26 | 8583399 | 8846705 | 263,307 | 45 | 3 | 2 |

**Table S5 Significant GO terms associated with genes in CNVRs**

| **Biological Process** | **GO name** | **Count** | **P value** | **Benjamini** |
| --- | --- | --- | --- | --- |
| GO:0009952 | anterior/posterior pattern specification | 27 | 4.2E-11 | 1.0E-7 |
| GO:0003002 | regionalization | 30 | 1.1E-9 | 1.3E-6 |
| GO:0001501 | skeletal system development | 38 | 5.0E-9 | 4.0E-9 |
| GO:0048706 | embryonic skeletal system development | 18 | 6.7E-9 | 4.0E-6 |
| GO:0007389 | pattern specification process | 32 | 8.8E-8 | 4.2E-5 |
| GO:0048598 | embryonic morphogenesis | 33 | 6.5E-7 | 2.6E-4 |
| GO:0009954 | proximal/distal pattern formation | 9 | 1.7E-6 | 5.9E-4 |
| GO:0043009 | chordate embryonic development | 31 | 2.4E-5 | 5.6E-3 |
| GO:0009792 | embryo development ending in birth or egg hatching | 31 | 2.8E-5 | 6.1E-3 |
| GO:0048562 | embryonic organ morphogenesis | 17 | 7.7E-5 | 1.5E-2 |
| GO:0030326 | embryonic limb morphogenesis | 13 | 1.6E-4 | 3.0E-2 |
| GO:0035113 | embryonic appendage morphogenesis | 13 | 1.6E-4 | 3.0E-2 |
| GO:0007265 | Ras protein signal transduction | 14 | 2.7E-4 | 4.5E-2 |
| GO:0048568 | embryonic organ development | 18 | 5.1E-4 | 7.8E-2 |
| GO:0035107 | appendage morphogenesis | 13 | 5.6E-4 | 7.9E-2 |
| GO:0035108 | limb morphogenesis | 13 | 5.6E-4 | 7.9E-2 |
| GO:0018101 | protein citrullination | 4 | 5.9E-4 | 8.0E-2 |
| GO:0006412 | translation | 55 | 4.1E-5 | 7.2E-2 |
| GO:0051726 | regulation of cell cycle | 54 | 8.0E-5 | 9.3E-2 |
| GO:0006414 | translational elongation | 27 | 1.7E-6 | 5.8E-3 |
| **Molecular Function** | **GO name** | **Count** | **P value** | **Benjamini** |
| GO:0043565 | sequence-specific DNA binding | 58 | 3.7E-9 | 2.8E-6 |
| GO:0030528 | transcription regulator activity | 93 | 6.0E-5 | 2.2E-2 |
| GO:0003700 | sequence-specific DNA binding  transcription factor activity | 66 | 6.3E-5 | 1.6E-2 |
| GO:0030554 | adenyl nucleotide binding | 186 | 1.6E-4 | 9.0E-2 |
| GO:0030546 | receptor activator activity | 10 | 7.4E-6 | 8.7E-3 |
| GO:0010861 | thyroid hormone receptor activator activity | 8 | 1.6E-5 | 9.5E-3 |
| GO:0030375 | thyroid hormone receptor coactivator activity | 8 | 1.6E-5 | 9.5E-3 |
| GO:0004886 | 9-cis retinoic acid receptor activity | 7 | 4.8E-5 | 1.9E-2 |
| GO:0030545 | receptor regulator activity | 12 | 5.4E-5 | 1.6E-2 |
| GO:0042809 | vitamin D receptor binding | 10 | 1.5E-4 | 3.4E-2 |
| GO:0030695 | GTPase regulator activity | 61 | 3.1E-4 | 5.9E-2 |
| GO:0060589 | nucleoside-triphosphatase regulator activity | 62 | 3.2E-4 | 5.3E-2 |
| GO:0003708 | retinoic acid receptor activity | 7 | 4.1E-4 | 5.8E-2 |
| **Cellular Componen** | **GO name** | **Count** | **P value** | **Benjamini** |
| GO:0043232 | intracellular non-membrane-bounded organelle | 141 | 2.7E-4 | 5.2E-2 |
| GO:0043228 | non-membrane-bounded organelle | 141 | 2.7E-4 | 5.2E-2 |
| GO:0030529 | ribonucleoprotein complex | 61 | 5.1E-5 | 2.7E-2 |
| GO:0042995 | cell projection | 72 | 6.8E-4 | 8.8E-2 |
| GO:0005840 | ribosome | 43 | 2.2E-6 | 1.3E-3 |
| GO:0030529 | ribonucleoprotein complex | 80 | 3.9E-6 | 1.2E-3 |
| GO:0022626 | cytosolic ribosome | 22 | 9.6E-6 | 1.9E-3 |
| GO:0044445 | cytosolic part | 31 | 4.9E-5 | 5.8E-3 |
| GO:0015934 | large ribosomal subunit | 18 | 8.9E-5 | 8.8E-3 |
| GO:0022625 | cytosolic large ribosomal subunit | 13 | 1.0E-4 | 7.5E-3 |
| GO:0070013 | intracellular organelle lumen | 205 | 3.8E-4 | 2.4E-2 |
| GO:0031974 | membrane-enclosed lumen | 212 | 4.5E-4 | 2.6E-2 |
| GO:0043233 | organelle lumen | 208 | 4.9E-4 | 2.6E-2 |
| GO:0005654 | nucleoplasm | 109 | 1.2E-3 | 5.8E-2 |
| GO:0005829 | cytosol | 169 | 1.0E-5 | 3.0E-3 |
| GO:0044445 | Cellular Component | 29 | 3.1E-4 | 3.6E-2 |
| GO:0022627 | cytosolic small ribosomal subunit | 12 | 7.6E-4 | 7.3E-2 |

**Table S6 Significant KEGG pathway associated with genes in CNVRs**

| Catagory | Term | Count | P-Value | Benjamin |
| --- | --- | --- | --- | --- |
| KEGG_ATHWAY | Ribosome | 36 | 5.3E-7 | 9.9E-5 |
| KEGG_PATHWAY | Noth signaling pathway | 20 | 2.0E-4 | 1.9E-2 |

Table S7 the commom CNVRs between Fontanesi and our study

| **ID** | **Chr** | **Start** | **End** | **Length** | **type** |
| --- | --- | --- | --- | --- | --- |
| 69 | 3 | 109244550 | 109499513 | 254963 | gain |
| 121 | 5 | 12337169 | 13599186 | 1262017 | loss |
| 146 | 6 | 78155315 | 80029344 | 1874029 | both |
| 180 | 9 | 13193845 | 16596810 | 3402965 | loss |
| 1801 | 9 | 13193845 | 16596810 | 3402965 | loss |
| 328 | 16 | 46846135 | 48389206 | 1543071 | gain |
| 3281 | 16 | 46846135 | 48389206 | 1543071 | gain |
| 374 | 18 | 64623786 | 66645322 | 2021536 | loss |
| 397 | 20 | 16642933 | 16980080 | 337147 | loss |
| 412 | 21 | 44468253 | 50055911 | 5587658 | loss |
| 429 | 24 | 19967 | 1380176 | 1360209 | loss |

Table S8: the commom CNVRs between Liu and our study

| **ID** | **Chr** | **Start** | **End** | **Length** | **type** |
| --- | --- | --- | --- | --- | --- |
| CNVR70 | 3 | 1057579 | 1379193 | 321615 | loss |
| CNVR79 | 3 | 207493246 | 207705614 | 212369 | loss |
| CNVR81 | 3 | 213058438 | 213286629 | 228192 | loss |
| CNVR92 | 5 | 12963601 | 13191989 | 228389 | loss |
| CNVR108 | 6 | 102654135 | 102875580 | 221446 | loss |
| CNVR113 | 7 | 20430110 | 20580046 | 149937 | loss |
| CNVR161 | 11 | 49921941 | 50038566 | 116626 | loss |
| CNVR187 | 16 | 46944599 | 47396344 | 451746 | loss |
| CNVR14 | 18 | 12221287 | 12500969 | 279683 | gain |
| CNVR213 | 21 | 44659875 | 45012897 | 353023 | loss |
| CNVR226 | 24 | 573924 | 696030 | 122107 | loss |
| CNVR5 | 17 | 24971029 | 26000884 | 1029856 | both |

Table S9 the commom CNVRs between Ma and our study

| **ID** | **Chr** | **Start** | **End** | **Length** | **type** |
| --- | --- | --- | --- | --- | --- |
| CNVR70 | 3 | 30394 | 4358207 | 321615 | loss |
| CNVR79 | 6 | 51437554 | 51934454 | 496900 | gain |
| CNVR81 | 10 | 5983222 | 6548405 | 565183 | gain |
| CNVR92 | 11 | 56630543 | 57068226 | 437683 | loss |
| CNVR108 | 3 | 1057579 | 1379193 | 321614 | loss |
| CNVR113 | 6 | 51867108 | 51900419 | 33311 | loss |
| CNVR161 | 10 | 6399268 | 6478156 | 78888 | loss |
| CNVR187 | 11 | 56774430 | 56911446 | 137016 | loss |

Table S10 All the primer sequences for qPCR

| CNV name | Forward primer | Reverse primer | Products |
| --- | --- | --- | --- |
| CNV86 | 5' CTGATGCATGAGTCTGGAGC 3' | 5' GGCCAGCAAAGAGTTTAACG 3' | 208bp |
| CNV190 | 5' TGTAGAGGTGACCTGAGCCA 3' | 5' CACTGCTCCTGTCACTTGGT 3' | 111bp |
| CNV233 | 5' CCCCATGCAGCTCGAGAA 3' | 5' GGGGCTCTTTCCTTGTGACA 3' | 134bp |
| CNV265 | 5' CAGGAGGCAACAGGAATCTA 3' | 5' GGCTGCTACTGTCTGAGAACTG 3' | 179bp |
| CNV338 | 5' GGGGACGACAGAGGATAAGA 3' | 5' GTCGTGTCTGACTCTTTGCG 3' | 138bp |
| CNV342 | 5' GGATCTGCTTCCCCCTCATA 3' | 5' ATGGATTTTGCTGGCTCTGA 3' | 135bp |
| CNV454 | 5' GTTTCCTCGGTGTGTATGCC 3' | 5' CGATCCAAAAATCTGCAAGC 3' | 190bp |
| CNV447 | 5' CTAGTCTTTGCCCTGCTTCA 3' | 5' GCTGAAGGTGATTGGTTCTATG 3' | 169bp |
| CNV58 | 5' CCCATTGGAGCTTTAATCACAT 3' | 5' CCAAGGCTGATACCTACGGA 3' | 223bp |
| CNV123 | 5' GCATCAATTCTTTGGCACTC 3' | 5' TGAAATTAAAAGACGTTTGCTC 3' | 183bp |
